# Supplementary material for: A tiny Triassic saurian from Connecticut and the early evolution of the diapsid feeding apparatus
Source: Nat Commun. 2018 Mar 23;9:1213. doi: 10.1038/s41467-018-03508-1 (PMC5865133; doi:10.1038/s41467-018-03508-1)
Supplement: Supplementary file 1 — Supplementary Information(PDF 2830 kb) [file 41467_2018_3508_MOESM1_ESM.pdf]

Supplementary Information for Pritchard *et al.*"A tiny Triassic saurian from Connecticut and the early evolution of the diapsid feeding apparatus"

Supplementary Note 1: Museum abbreviations used in comparative description/phylogenetic analysis

AMNH–American Museum of Natural History (New York, NY, U.S.A.)
BP–Evolutionary Studies Institute, University of Witwatersrand (Johannesburg, South Africa)
CAS–California Academy of Sciences (San Francisco, CA, U.S.A.)
CM–Carnegie Museum of Natural History (Pittsburgh, PA, U.S.A.)
GMPKU–Geological Museum of Peking University (Beijing, China)
GR–Ruth Hall Museum of Paleontology (Abiquiu, NM, U.S.A.)
IVPP–Institute of Vertebrate Paleontology and Paleoanthropology (Beijing, China).
MCSN–Museo Civico di Storia Naturali Milano (Milano, Italy)
MCSNB–Museo Civico di Scienze Naturali Enrico Caffi (Bergamo, Italy)
MCZ–Museum of Comparative Zoology (Cambridge, MA, U.S.A.)
MFSN–Museo Friulano di Storia Naturale (Udine, Italy)
MNHN–Muséum National d'Histoire Naturelle (Paris, France)
MPUM–Museo di Paleontologia Università di Milano (Milano, Italy)
NHMUK–Natural History Museum of the United Kingdom (London, UK)
NMMNH–New Mexico Museum of Natural History (Albuquerque, NM, U.S.A.)
NMQR–National Museum Bloemfontein (Bloemfontein, South Africa).
PIMUZ–Paleontological Institut und Museum (Zürich, Switzerland)
PIN–Paleontological Institute (Moscow, Russia)
PVSJ–Museo de Ciencias Naturales, Universidad Nacional de San Juan (San Juan, Argentina).
RC–Rubidge Collection (Wellwood, Graaff-Reinet, South Africa)
SAM PK–Iziko Museum (Cape Town, South Africa)
SMNS–Staaliches Museum für Naturkunde Stuttgart (Stuttgart, Germany)
TMM–Texas Memorial Museum (Austin, TX, U.S.A.)
UA–Université d'Antannanarivo (Antannanarivo, Madagascar)
USNM PAL – Paleobiology collections of United States National Museum of Natural History (Washington, DC, U.S.A.).
USNM VZ – Vertebrate Zoology collections of United States National Museum of Natural History (Washington, DC, U.S.A.).
WMsN–Westfäliches Museum für Naturkunde, Münster (Münster, Germany)
YPM R– Herpetology collections of Yale Peabody Museum of Natural History (New Haven, CT, U.S.A.)
YPM VP– Vertebrate Paleontology collections of Yale Peabody Museum of Natural History (New Haven, CT, U.S.A.)
ZPAL–Institute of Paleobiology, University of Warsaw (Warsaw, Poland)

Supplementary Note 2: Taxon list for comparative anatomical description and phylogenetic analysis

Bibliographic references and institutional accession numbers of specimens that were scored based on firsthand examination.

Colobops noviportensis, gen. et sp. nov.–YPM VPPU 18835, references [1,2].

Non-saurian Diapsida
Acerosodontosaurus piveteaui–[3], [4].
Avicranium renestoi–AMNH FARB 30834.
Claudiosaurus germaini–CM 47510; MNHN MAP 1; SAM K8263, K8266; [5,6].
Coelurosauravus elivensis–MNHN IP 1908-11-21, 1908-11-22, 1908-5-2; [7–9]
Dolabrosaurus aquatilis–CM 28589; [10].
Drepanosaurus unguicaudatus–MCSNB 5728; [11,12].
Hovasaurus boulei–MNHN MAP 336, [6,13].
Hypuronector limnaios–AMNH FARB 1721, 7759; [14].
Megalancosaurus preonensis–MFSN 1721; MPUM 6008, 8437; [11].
Petrolacosaurus kansensis–CM 29904; [15].
Rautiania spp.–dozens of specimens curated at PIN, catalogued in references [9,16].
Thadeosaurus colcapani–MNHN MAP 360; [5,17].
Tropidostoma Zone younginiform–SAM PK 7710, 8565, 10818; [18].
Vallesaurus cenensis–MCSNB 4751; [11].
Weigeltisaurus jaekeli–SMNS 53349, ; [7,8,19,20].
Youngina capensis–AMNH FARB 5561; BP/1 375, 2871, 3859; SAM PK 7578, 10777; [2113,22,23].

Non-archosauriform pan-Archosauria
Amotosaurus rotfeldensis– SMNS 50691, partial skull and anterior portion of skeleton; 53783, multiple associated skeletons; 54784, two skulls, one with associated neck; 54810, dissociated skeleton; 90600, sacrum and partial tail; 90601, articulated maxilla and jugal; [24].
Azendohsaurus laaroussii– Collections of maxillae and dentaries on hand at MNHN. A single premaxilla (MNHN ALM 365-16).
Azendohsaurus madagaskarensis–Hundreds of specimens accessioned with Université d'Antannanarivo and the Field Museum of Natural History; [25], [26]
Bentonyx sidensis–[27,28]
Boreopricea funerea–PIN 3708/1, 3708/2; [29].
Brasinorhynchus mariantensis–[30]
Eohyosaurus woolvardti–Butler et al., 2015.
Howesia browni–SAM PK-5884, 5885, 5886; [31].
Juvenile hyperodapedontine rhynchosaur (formerly identified as *Scaphonyx fischeri*)–MCZ 1664, [32].
Icarosaurus siefkeri–AMNH FARB 2101; [33].
Kuehneosaurus latus–AMNH FARB 7761–7798, NHMUK R 5972, 5975, 5976, 5979, 5982, 5985, 5987, 5988–5990, 5992, 5994, 6001, 6002, 6012, 6015, 6021, 6027, 6030, 6059, 6060, 6069, 6073, 6076, 6077, 6086, 6179, 6182, 6205, 8172 (holotype), 12783, 12874, 12877, 12880, 12894, 12895, 12900, 12902, 12903; [34,35].
Langobardisaurus pandolfii–MCSNB 2883, 4860; MFSN 1921; [36].
Macrocnemus bassanii–MCSN BES SC 111, V 457; PIMUZ T.2472, 2477, 4355, 4822; [37,38].
Macrocnemus fuyuanensis–GMPKU P-3001, [39].
Mesosuchus browni–SAM PK-5882, 6046, 6536, 7416, 7701; [40]; CT scan data of SAM-PK 6536.

Pamelaria dolichotrachela–[41,42].
Protorosaurus speneri–NMNH 442453 (paleo.); SMNS cast of WMsN P 47361; [43].
Rhynchosaurus articeps–NHMUK R 1236, 1237, 1239; [44,45]
Tanystropheus longobardicus–MCSN BES SC 265, 1018, V 3730; PIMUZ T/1277, T/2819; [46,47].
Tanytrachelos ahynis–AMNH FARB 7206; VMNH 2826, 3423, 120015, 120016, 120019, 120042, 120043, 120046, 120047, 120048, 120049; YPM VP 7482, 8600; [48,49].
Teraterpeton hrynewichorum–[50].
Teyujagua paradoxa–[51]
Teyumbaita sulcognathus–[52,53].
Trilophosaurus buettneri–Several dozen specimens in the collections of TMM, primarily TMM 31025-140; [26,54,55].
Trilophosaurus jacobsi–Several dozen specimens in the collections of NMMNH (for skull data, primarily NMMNH P-41400); [56].

<u>Archosauriformes</u>
Batrachotomus kupferzellensis–Cranial and postcranial elements at SMNS; [57–59].
Chanaresuchus bonapartei–MCZ 4039; [60,61].
"*Chasmatosaurus*" *yuani*–IVPP V4067, casts of IVPP V36315; [62,63].
Coelophysis bauri–[64,65].
Erythrosuchus africanus–NHMUK R3592, NMQR 3765; [66–68].
Euparkeria capensis–SAM PK 7696; [69,70].
Gracilisuchus stipanicicorum–MCZ 4117.
Herrerasaurus ischigualastensis–Digimorph scans of PVSJ 407.
Melanorosaurus carinatus–[71]
Plateosaurus engelhardti–[65,72,73]
Proterosuchus spp. (containing the South African proterosuchid species)–BP/1 3393, 4601; NMQR 880, 1484; CT scan data of RC 846 (part of [74]; SAM PK10603; [75,76].
Shansisuchus shansisuchus–[77]

<u>Lepidosauria</u>
Clevosaurus brasiliensis–[78,79].
Clevosaurus hudsoni–NHMUK PLR 600, 605, 606, 608, 615–617, 621, 622, 625, 676, 677, 716, 733, 787, 790, 809, 811, 818; NHMUK R 36832; [80]; [81].
Ctenosaura similis–YPM R 10533.
Diphydontosaurus avonis–MCSNB 4862; NHMUK R 16101, 16520, 36997; [82].
Dracaena guianensis–YPM R 11075, 11286, 12496.
Gephyrosaurus bridensis–NHMUK T 952, 1548, 1819, 1863, 1864, 2045, 2082 ; [83–85].
Hydrosaurus pustulatus–USNM VZ 78168, 78170, 497579–497581.
Iguana iguana–USNM VZ 70470, 220231, 220232, 220236, 248781; YPM R 10869, 11188, 11622, 13952, 18330.
Physignathus lesueurii–YPM R 11121, 12137, 16672, 16779.
Planocephalosaurus robinsonae–[81,86,87].
Pogona vitticeps–YPM R 12139, 16629.
Sauromalus varius–YPM R 19209, 19211.
Sceloporus occidentalis–YPM R 17142, 17158, 17160.
Sceloporus undulatus–YPM R 15006.

Shinisaurus crocodilurus–[88,89].
Sphenodon punctatus–YPM R 10646, 11431; CAS 208882; [90–9899].
Teius teyou–YPM R 13709, 13710, 13938.
Uromastyx acanthinura–YPM R 13525.
Varanus exanthematicus–YPM R 11187, 13940, 19118.
Varanus komodensis–YPM R 10881, 16943.

Supplementary Note 3: Characters for phylogenetic analysis

The phylogenetic dataset used here is a modification of the matrix initially used in Pritchard et al.[49], incorporating subsequent modifications in Nesbitt et al.[26] and Pritchard and Nesbitt[100]. **Bolded** characters have been modified in a substantial way from their use in Pritchard and Nesbitt[100].

1) Premaxilla external sculpturing: (0) surface is smoothly sculptured, (1) premaxilla is marked by anteroventral striations.
2) Premaxilla, ventral margin, orientation relative to long axis of skull: (0) margin horizontal, roughly inline with maxillary ventral margin; (1) slight downturn, such that the margin trends anteroventrally; (2) extensive downturn, premaxilla extends to ventral margin of dentary. ORDERED.
3) Premaxilla, anterodorsal process (=nasal process): (0) present, separating the nares; (1) absent or reduced.
4) Premaxilla, posterodorsal process (=maxillary process, subnarial process): (0) absent, such that premaxilla contributes a small ventral margin for the external naris; (1) posterodorsal process present, framing the posteroventral margin of the external naris.
5) Premaxilla, posterodorsal process (=maxillary process, subnarial process), length: (0) short, failing to exclude maxilla from narial margin; (1) long, excluding maxilla from narial margin; (2) extremely long, reaching the anteriormost part of the prefrontal. ORDERED.
    - This character was not ordered in [100]. As the states represent a transformational series with nested intermediates, we have ordered it here.
6) **Premaxilla, posterodorsal process, maxilla contact: (0) simple, straight suture; (1) margin/knob on the posterior margin of the posterodorsal process of the premaxilla fits into notch in the anterior surface of the maxilla; (2) anterior lamina of maxilla laps laterally over posterodorsal process of premaxilla.**
    - Our interpretations of these states are illustrated in Figures S10 and S11. State 2 was added for this analysis, describing the condition in YPM VPPU 18835 and Rhynchosauria (e.g., *Mesosuchus browni*, SAM-PK 6536; *Rhynchosaurus articeps*, NHMUK R1237).
7) Maxilla, ventral margin, shape: (0) horizontal, (1) convex.
8) Maxilla, posterolateral surface: (0) directly adjacent to alveolar margin, (1) lateral process of maxilla present, creating distinct space between maxillary alveoli and posterolateral surface of the maxilla.
9) Nasal, contact with prefrontal, orientation: parasagittal, (1) oriented anterolateral.

10) Maxilla, lateral surface near anteroposterior midpoint: (0) marked by subequal neurovascular foramina, (1) bears single neurovascular foramen that is anteroposteriorly longer than all others.
11) Lacrimal, facial contribution: (0) forms a portion of lateral surface of the face that reaches anteriorly to the external naris; (1) forms a portion of the lateral surface of the face but does not reach external naris; (2) limited to orbital margin. ORDERED.
12) Lacrimal, facial contribution, dorsal portion: (0) extends dorsally to reach the ventral margin of the nasal; (1) externally, lacrimal fails to reach nasal.
13) Antorbital fenestra: (0) absent, (1) present;
14) Frontal, fusion to contralateral frontal: (0) unfused, suture patent; (1) fused in the midline, no clear suture dorsally.
15) Frontals, shape: (0) maintains transverse width throughout its anteroposterior length; (1) gradual transverse expansion towards posterior margin of bone; (2) abrupt transverse expansion in postorbital region; (3) tapered posteriorly due to transverse breadth of postfrontals.
16) Frontal, shape of contact with parietal: (0) roughly transverse in orientation; (1) Anteriorly convex, U-shaped contact, with frontal exhibiting posterolateral processes at contact.
17) Frontal and postfrontal, dorsal surfaces, texture: (0) relatively smooth; (1) distinct pitting.
18) Postfrontal: (0) present, (1) absent as discrete ossification.
19) Parietal, fusion to contralateral parietal: (0) unfused to one another, patent suture; (0) fused at the midline, no distinct suture.
20) Parietal, dorsal surface: (0) flattened skull table; (1) dorsal exposure of parietal forms a raised margin elevated above lateral excavation for jaw adductor musculature; (2) thin blade like sagittal crest. ORDERED.
21) Parietal, posterolateral (=post-temporal, supratemporal) processes, orientation: (0) roughly transverse, (1) angled strongly posterolaterally.
22) Pineal foramen: (0) present, (1) absent.
23) Pineal foramen, position on skull roof: (0) entirely surrounded by parietals, (1) situated within the frontoparietal suture.
24) Postparietals: (0) absent as discrete ossifications; (1) present.
25) Postparietal, fusion in midline: (0) unfused, with patent suture evident; (1) fused as a midline interparietal.
26) Postorbital, medial process: (0) absent, with contributions of the frontal, parietal, and/or postfrontal forming the posterodorsal orbital margin; (1) present, postorbital contributing to posterodorsal orbital margin.
27) Postorbital, medial contact with frontal and parietal: (0) present, (1) absent with postfrontal fitted in between.
28) Postorbital, posterior process, anteroposterior length: (0) contributes to lateral margin of supratemporal bar, but does not reach the posterior aspect of the infratemporal fenestra; (1) contributes to the entire anteroposterior length of the supratemporal bar reaching the posterior aspect of the infratemporal fenestra.
29) Infratemporal fenestrae, conformation: (0) present as distinct opening, framed by squamosal, postorbital, and jugal; (1) postorbital, jugal, and squamosal fit against one another as a lateral temporal plate.

30) Jugal, lateral surface, ornamentation: (0) unornamented; (1) distinct anteroposteriorly trending shelf present.
31) Jugal, dorsal process, contact with squamosal: (0) absent, (1) present.
32) Jugal, posterior process: (0) absent, (1) present but failing to contact the quadratojugal posteriorly; (2) present, contacting the quadratojugal posteriorly. ORDERED.
33) Squamosal, lateral (=descending) process/flange: (0) anteroposteriorly broad, covering the quadrate entirely in lateral view; (1) anteroposteriorly slender, partially exposing quadrate; (2) absent. ORDERED.
34) Squamosal, posterior lamina: (0) present, covering much of posterior aspect of quadrate; (1) absent, posterior aspect of quadrate exposed in occipital view.
35) Squamosal, contact with quadrate: (0) braces quadrate laterally; (1) dorsal portion of bone forms broad contact with dorsal surface of quadrate.
36) Supratemporal: (0) absent as discrete ossification: (1) present.
37) Tabulars (0) absent, (1) present.
38) Quadratojugal: (0) present, (1) absent as distinct ossification.
39) Quadratojugal, anterior process: (0) present, (1) absent.
40) Quadratojugal, anterior process, shape: (0) paralleling dorsal and ventral borders, (1) anteriorly tapering anterior process.
41) Quadrate, posterior margin, shape: (0) straight, vertically oriented; (1) concave, excavated.
42) Quadrate, lateral flange (=tympanic crest): (0) absent, quadrate has no lateral expansion; (1) present.
43) Quadrate, tympanic crest, conch: (0) absent, (1) present as deep concavity on posterior surface of crest.
44) Palatal teeth: (0) present, (1) absent.
45) Vomer, teeth: (0) present, (1) absent.
    - Taxa coded as “1“ for character 44 are coded as “-”for this character.
46) Vomer, contact with maxilla: (0) absent, vomer only contacts premaxilla; (1) present, vomer premaxilla contact expands onto maxilla.
47) Palatine teeth: (0) present, (1) absent.
    - Taxa coded as “1“ for character 44 are coded as “-”for this character.
48) Palatine. lateral tooth row, dental morphology: (0) similar to other palatal teeth; (1) enlarged relative to all other palatal teeth, akin to marginal teeth in size and morphology.
49) Pterygoid, anterior process dentition, medial row (row T3 of Welman, 1998 & Ezcurra, 2016): (0) absent, (1) present.
50) Pterygoid, anterior process dentition, lateral row (row T2 of Welman, 1998 & Ezcurra, 2016): (0) absent, (1) present.
51) Pterygoid, transverse process, dentition: (0) absent, (1) present.
52) Pterygoid, transverse process, dentition, number of tooth: (0) multiple rows (1) one row.
53) Pterygoid, midline contact with contralateral pterygoid: (0) absent, (1) present, small contact present at anterior tips; (2) present, broad contact throughout anteroposterior length. ORDERED.
54) Pterygoid, transverse process, orientation of long axes: (0) lateral, (1) anterolateral.
55) Supraoccipital, posterior surface: (0) smooth; (1) distinct dorsoventrally running crest in the midline.

56) Supraoccipital, shape: (0) consists of a flattened posterior lamina, (1) pillar like (U-shaped in dorsal view).
57) Opisthotic, ventral ramus, shape: (0) slender process, (1) distinct club shaped expansion ventrally.
58) Opisthotic, paroccipital process, contact with suspensorium: (0) absent, ends freely; (1) present.
59) Exoccipital, contact with dorsal elements of occiput: (0) exoccipitals columnar throughout their dorsoventral height, forming transversely narrow contact with dorsal occiput elements; (1) exoccipitals exhibit dorsomedially inclined processes which do not meet in the midline; (2) exoccipitals meet dorsally over the foramen magnum excluding the supraoccipital from that opening. ORDERED.
60) Exoccipital, contact on floor of foramen magnum with contralateral exoccipital: (0) absent, basioccipital contributes to floor of foramen magnum; (1) present, excluding basioccipital from floor of the foramen magnum.
61) Exoccipital, fusion with other braincase elements: (0) unfused to other braincase elements, sutures with basioccipital and opisthotic patent; (1) exoccipital fused to opisthotic; (2) exoccipital fused to basioccipital.
62) Opisthotic, paroccipital process, morphology: (0) unflattened and tapered, (1) anteroposteriorly flattened distally.
63) Basioccipital, occipital condyle, posterior surface: (0) exhibits elliptical notochordal depression that occupies much of posterior surface of condyle; (1) exhibits narrow “pinprick” notochordal pit within posterior surface; (2) condyle is smoothly convex. ORDERED.
64) Basioccipital, basal tubera: (0) poorly developed, not extending well ventral of occipital condyle; (1) well developed, extending ventral to level of occipital condyle.
65) Parabasiphenoid, cultriform process, dentition: (0) absent, (1) present;
66) Parabasisphenoid, parasphenoid crests: (0) absent, such that there is no ventral floor for the vidian canal, (1) present as prominent ventrolateral extensions of the caudoventral processes, framing the ventromedial floor of the vidian canal.
67) Parabasisphenoid, passage for internal carotid arteries: (0) within lateral wall of braincase, (1) within ventral surface of the parabasisphenoid, (2) passage of the internal carotids do not enter the braincase.
68) Parabasisphenoid, conformation of ventral surface: (0) roughly planar, (1) distinct depression at the suture between the basioccipital and the parabasisphenoid, (2) distinct depression within the parabasisphenoid.
69) Parabasisphenoid, cultriform process: (0) extremely elongate, reaching to the level of the internal nares; (1) shorter, failing to reach internal nares.
70) Parabasisphenoid, basipterygoid process, orientation of long axes: (0) anterolateral, (1) lateral.
71) Parabasisphenoid, abducens foramina: (0) within the dorsum sella, (1) track across dorsal surface of dorsum sella.
72) Laterosphenoid ossification: (0) absent; (1) present, but fails to reach ventral surface of frontals; (2) present reaching ventral surface of frontals. ORDERED.
73) Prootic, lateral surface, anteroventrally oriented crest (=crista prootica): (0) present, (1) absent.

74) Prootic, anteroventral surface, anterior inferior process: (0) present, framing anterior margin of trigeminal foramen; (1) absent, trigeminal foramen unframed anteriorly.

75) Prootic, posterolateral surface, contribution to paroccipital process: (0) absent, no contribution to anterior surface of paroccipital process; (1) present, contributes laterally tapering lamina to the anterior surface of the process.

76) Stapes, dorsal process: (0) absent, (1) present.

77) Stapes, foramen for stapedial artery: (0) present, (1) absent.

78) Dentary, anterior portion, symphyseal region of mandible: (0) dentaries do not diverge, (1) tips of dentaries diverge from one another.

79) Coronoid process: (0) absent, (1) present.

80) Surangular, lateral surface, foramen positioned near surangular-dentary contact: (0) absent, (1) present.

81) Surangular, lateral surface, foramen positioned directly anterolateral to glenoid fossa: (0) absent, (1) present.

82) Angular, exposure on lateral mandibular surface: (0) broadly exposed, (1) limited to <1/3 the dorsoventral height of the mandible.

83) Angular, exposure on lateral mandibular surface: (0) terminates anterior to the glenoid, (1) extends to the glenoid.

84) External mandibular fenestra (EMF): (0) absent, (1) present.

85) Splenial, contribution to mandibular symphysis: (0) splenials contribute to symphysis, (1) splenials fail to contribute.

86) Retroarticular process: (0) present as extension of articular and adjacent bones posterior to quadrate articulation, (1) absent.
    - Taxa code as “1” for this character are coded as “-“ for character 266.

87) Articular, fusion to prearticular: (0) absent, (1) present.

88) Marginal dentition on anteriormost portions of premaxilla and dentary: (0) present, (1) absent.

89) Marginal dentition, enlarged caniniform teeth in maxilla: (0) present, (1) absent, maxillary teeth subequal in size.

90) Marginal dentition, serrations: absent (0), present (1).

91) Marginal dentition, posterior margin of tooth, shape: (0) convex or straight, (1) concave.

92) Marginal dentition, arrangement on dentigerous surface of maxilla: (0) single row of marginal teeth, (1) multiple zahnreihen in maxilla.

93) Marginal dentition, morphology of crown base: (0) single pointed crown, (1) flattened platform with pointed cusps, (2) mesiodistally arranged cusps.

94) Marginal dentition, implantation: (0) teeth situated in shallow groove (as in pleurodonty, thecodonty); (1) teeth superficially attached to tooth bearing bones, with limited extension of pulp cavity into the bone teeth; (2) superficially attached to tooth bearing bones, with no extension of pulp cavity into the bone (= true acrodonty). ORDERED.
    - This character was not ordered in [100]. As the states form a logical series with intermediates, we have ordered it for this analysis.

95) Marginal dentition, lingual surface: (0) teeth walled by minimal lingual wall, (1) no lingual wall (=pleurodonty).

96) Marginal dentition, lingual surface: (0) teeth walled only by minimal lingual wall, (1) interdental plates are present.

97) Marginal dentition, rooting: (0) tooth crowns are not attached to dentigerous bones teeth, (1) ankylosed to bones of attachment.
98) Marginal dentition, tooth shape at crown base: (0) subcircular, (1) labiolingually compressed, (2) labiolingually wider than mesiodistally long.
99) Marginal dentition, regionalization: (0) no clear abrupt change from anterior to posterior teeth, (1) clear shift from small anterior juvenile teeth anteriorly to large posterior adult teeth.
100) Marginal dentition, procumbency: (0) anteriormost marginal teeth have similar apicobasal orientation to posterior teeth, (1) anteriormost teeth are procumbent.
101) Vertebrae notochordal canal: (0) present, (1) absent.
102) Presacral vertebrae, posterior articular surface: (0) planar, (1) concave, (2) convex.
103) Presacral vertebrae, posterior convexity: (0) slight with posteriorly flattened surface; (1) strong, hemispherical.
  - Taxa coded as “0” or “1” for character 102 are coded as “-” for this character.
104) Anterior cervical ribs, shaft, shape: (0) tapering rapidly, roughly triangular in lateral view; (1) ribs taper gradually, elongate and splint like in lateral view.
105) Cervical ribs, anterior process: (0) absent, (1) present.
106) Intercentra in the cervical region: (0) present, (1) absent.
107) Anterior post axial cervical vertebrae, shape of anterior articular surface: (0) subcircular, roughly equivalent in dorsoventral height and transverse width; (1) compressed with a greater transverse width than dorsoventral height.
108) Cervical vertebrae, ventral keel: (0) present, (1) absent.
109) Anterior post axial cervical vertebrae, ventral surface, shape excluding keel: (0) convex, rounded; (1) flattened.
110) Cervical vertebrae, costal facet, number: (0) one, (1) two.
111) Anterior post axial cervical vertebrae, position of diapophysis or dorsal margin of synapophyses: (0) at or near dorsoventral level of pedicles, (1) further ventrally near the dorsoventral midpoint of the centrum.
112) Anterior post axial cervical vertebrae, costal facets, position relative to one another: (0) distinctly offset from one another, (1) facets very closely appressed to one another with little or no finished bone separation.
113) Anterior post axial cervical vertebrae, neural spine, shape of base: (0) anteroposteriorly elongate, subequal in length to the neural arch; (1) short spine restricted to posterior half of neural arch.
114) Anterior post axial cervical vertebrae, neural spine, shape in cross section: (0) transversely narrow, (1) elliptical or circular.
115) Anterior post axial cervical vertebrae, neural spine, anterior margin, shape: (0) straight and linear; (1) anterodorsal process present, forming an anterior notch.
116) Anterior post axial cervical vertebrae, neural spine, anterior margin, inclination: (0) posterodorsal, (1) anterodorsal.
117) Anterior post-axial cervical vertebrae, neural spine, dorsal tip: (0) transversely slender, (1) expanded transversely.
118) Mid-cervical vertebrae, neural spine, height: (0) equivalent in height and length to other cervical neural spines; (1) dorsoventrally depressed at anteroposterior midpoints, leaving them little more than midline dorsal ridges.

119) Cervical vertebrae, postzygapophyses, dorsal surfaces: (0) smooth and rounded, marked by dorsoventrally tall projections (=epipophyses).

120) Anterior dorsal vertebrae, position of parapophysis or ventral margin of dorsal portion of synapophysis: (0) partially on lateral margin of centrum, (1) entirely on neural spine.

121) Posterior dorsal vertebrae, position of parapophysis or ventral margin of dorsal synapophysis: (0) partially on lateral margin of centrum, (1) positioned entirely on neural spine.

122) Anterior dorsal vertebrae, pectoral region, number of costal facets: (0) one (=holocephaly), (1) two (=dichocephaly), (2) three (=tricephaly). ORDERED.
   - This character was not ordered in [100]. As the states here form a logical series with intermediate steps, we have ordered it for this analysis.

123) Posterior dorsal vertebrae, costal facets: (0) single rib facet, (1) inverse L rib facet, suggesting partial confluence of diapophysis and parapophysis; (2) double rib facet.

124) Posterior dorsal vertebrae: (0) ribs fused to costal facets, (1) unfused.

125) Dorsal vertebrae, neural spines, dorsal portion: (0) similar width as the more distal portion of the neural, (1) spine expanded transversely into a flattened tip spine table.

126) Dorsal vertebrae, neural arches, dorsolateral surfaces: (0) marked by rounded mammillary processes, (1) smooth.

127) Dorsal vertebrae, neural spine, dorsal tip, texturing: (0) marked by pebbly unfinished bone, (1) marked by transverse striations of bone.
   - Taxa coded as “0” for character 125 are coded as “-“ for this character.

128) Dorsal vertebrae, intercentra: (0) present, (1) absent.

129) Dorsal vertebrae, neural spines, dorsoventral height: (0) tall, greater in dorsoventral height than anteroposterior length; (1) long and low, lesser in dorsoventral height than anteroposterior length.

130) Dorsal vertebrae, accessory zygosphene zygantrum articulations: (0) absent, (1) present.

131) Second sacral rib, shape: (0) rib is a single unit, (1) rib bifurcates distally into anterior and posterior processes.

132) Second sacral rib, posterior process: (0) terminally blunted, (1) sharp distally.
   - Taxa coded as “0” for Character 131 are coded as “-“ for this character.

133) Anterior caudal vertebrae, transverse processes, shape: (0) curve posterolaterally, (1) straight, (2) curved anterolaterally.

134) Anterior caudal vertebrae, transverse processes, medial base, orientation: (0) perpendicular to the long axis of the vertebra, (1) angled posterolaterally.

135) Caudal vertebrae, autotomic septa within centra: (0) absent, (1) present.

136) Chevrons, hemal spine, shape: (0) tapers along its proximodistal length; (1) broadens slightly along its length; (2) broadens distally, forming inverted T shape broadens distally forming subcircular expansion.

137) Chevrons, hemal spine, length: (0) similar in length or shorter than caudal neural spines, (1) substantially longer than caudal neural spines.

138) Chevrons, hemal spine, curvature: (0) roughly straight, (1) convex anteriorly.

139) Epiphyses of limb elements secondary ossification centers: (0) absent, (1) present.

140) Cleithrum: (0) present, (1) absent.

141) Clavicle, ventral articular portion: (0) broader anteroposteriorly than distal portion of clavicle, (1) similar in anteroposterior narrowness to the distal portion of the clavicle.

142) Interclavicle, anterior portion of bone: (0) transversely robust, forming broad diamond; (1) transversely gracile, forming slender, anchor like shape anteriorly.
143) Interclavicle, anterior surface between clavicular articulations: (0) smooth margin, (1) prominent notch in margin.
144) Interclavicle, posterior stem, shape: (0) slender, tapering; (1) marked transverse expansion.
145) Scapula, scapular blade, shape: (0) flattened blade directed dorsally, (1) flattened blade with large posterior concavity; (2) anterodorsally curved blade.
146) c: (0) bears prominent tubercle; (1) smooth bone, lacking tubercle.
147) Coracoid ossifications, number: (0) two, (1) one.
148) Coracoid, infraglenoid morphology: (0) no development of coracoid posteroventral to glenoid, (1) prominent post glenoid process on coracoid, terminating in thickened margin.
149) Sternum, ossification of sternal plates: (0) absent, (1) present.
150) Humerus, ectepicondyle, radial nerve groove: (0) absent, (1) present.
151) Humerus, ectepicondyle, radial nerve groove: (0) no roof, (1) roof present, forming ectepicondylar foramen.
152) Humerus, ectepicondyle: (0) present as prominent preaxial crest, (1) absent, no crest.
153) Humerus, entepicondylar foramen: (0) entepicondylar foramen absent, (1) entepicondylar foramen present.
154) Humerus, entepicondyle, morphology: (0) smooth margin between shaft and post axial condyle, (1) prominent entepicondylar crest present.
155) Humerus, entepicondylar crest, proximal margin, morphology: (0) crest exhibits a curved proximal margin, (1) crest exhibits a prominently angled proximal margin.
156) Humerus, distal condyles, morphology: (0) distinct trochlear and capitular articulations, (1) low double condyle.
157) Ulna, ossified olecranon process: (0) present, (1) absent.
158) Medial centrale of manus: (0) absent, (1) present.
159) Distal carpal five: (0) absent, (1) present.
160) Manual intermedium: (0) present, (1) absent.
161) Ulnare and intermedium, perforating foramen between elements: (0) present, (1) absent.
162) Manual digit four, phalangeal formula: (0) five phalanges, (1) four phalanges.
163) Pelvis, puboischiadic plate, fenestration: (0) no fenestra, (1) thyroid fenestra within plate.
164) Ilium, iliac blade, long axis, orientation: (0) horizontal orientation, (1) posterodorsal orientation, (2) anterodorsal orientation.
165) Ilium, anteroventral process extending from anterior margin of pubic peduncle: (0) absent, (1) present, process draping across anterior surface of pubis.
166) Ilium, supra acetabular crest (0) absent, posterodorsal margin of acetabulum similar in development of anterodorsal margin, (1) prominent anterodorsal bony lamina frames the anterodorsal margin of the acetabulum.
167) Ilium, supra-acetabular surface: (0) dorsalmost margin of acetabulum is unsculptured, (1) prominent bulbous rugosity superior to acetabulum.
168) Ilium, acetabulum, lateral surface: (0) irregular, marked by posterodorsal invasion by finished bone; (1) roughly circular, no posterodorsal invasion.
169) Ilium, iliac blade, anterior surface: (0) smooth anterior margin (1) anteriorly projecting process or tuber present.

170) Ilium, anterior process/tuber: (0) small, with anterodorsal margin of ilium curving smoothly into dorsal margin of iliac blade; (1) large and anteriorly projecting, with dorsal margin of tuber nearly continuous with dorsal margin of iliac blade.

171) Ilium, posterior process, anteroposterior length: (0) weakly developed failing to extend well posterior of acetabulum, (1) strongly developed extending well posterior to the acetabulum.

172) Ilium, iliac blade, dorsal margin: (0) smoothly textured dorsal border, (1) marked by distinct dorsoventral striations running from acetabulum to dorsal margin of iliac blade.

173) Pubis, symphysis: (0) pubic apron present with distinct anteroventral downturn of the symphyseal region; (1) pubic apron absent, symphysis sits only in coronal plane.

174) Pubis, pubic tubercle: (0) absent, with anterolateral surface of pubis unexpanded; (1) present, with anterolateral surface of pubis expanded into anteroposteriorly broadened tuber.

175) Pubis, anterolateral surface: (0) lateral pubic tubercle (sensu [101] present, manifesting as rounded tuberosity; (1) prominent, transversely narrow ambiens flange present; (2) anterolateral surface of pubis marked by rugose bone.

176) Ischium, posterior margin: (0) vertical and flattened, (1) posterior process extends from posterodorsal margin of bone (*spina ischii* sensu [102].

177) Femur, profile in pre axial view: (0) sigmoidal curvature, (1) linear shaft with slight ventrodistal curvature.

178) Femur, proximal surface: (0) well ossified, convex; (1) concave surface with central groove.

179) Femur, internal trochanter, proximal portion: (0) crest does not reach femoral head; (1) crest reaches far proximally, continuous with proximal articular surface.

180) Femur, distal condyles, relative size: (0) medial and lateral condyles subequal in transverse/proximodistal dimensions; (1) condyles unequal, lateral condyle larger than the medial condyle.

181) Femur, distal condyles, dimensions relative to femoral shaft: (0) distinct expansion beyond the circumference of the femoral shaft, (1) limited expansion beyond the circumference of the femoral shaft;

182) Femur, tibial condyle: (1) medial surface is rounded and mound like, (1) medial surface is triangular and sharply pointed.

183) Femur, fibular condyle, ventral surface: (0) flattened and planar, (1) rounded and mound like.

184) Pedal centrale: (0) absent as distinct ossification, (1) present as distinct ossification.

185) Proximal tarsals (astragalus and calcaneum), co-ossification: (0) present as distinct ossifications, (1) co-ossified.

186) Proximal tarsals (astragalus and calcaneum), perforating foramen: (0) present, situated between astragalus and calcaneum, (1) absent.

187) Calcaneum, distal facet: (0) little broader in dorsal-plantar dimensions than proximal facet; (1) distal facet is markedly expanded in dorsal-plantar dimensions, more than twice the breadth of the proximal facet.

188) Calcaneum, lateral margin: (0) terminates in unthickened margin, (1) roughened tuberosity present laterally.

189) Calcaneum, lateral margin, transverse dimension: (0) little postaxial expansion; (1) markedly broadened, lateral wing of calcaneum twice as broad or broader than the distal calcaneal facet.
190) Calcaneum, lateral projection, ventrolateral margin: (0) coplanar with dorsolateral margin of projection, (1) ventrolateral margin of calcaneum curls externally.
191) Distal tarsal four, proximal surface: (0) smooth contact surface for proximal tarsals, (1) prominent process for contact with proximal tarsals.
192) Pedal centrale, contact with tibia: (0) absent, (1) present.
193) First distal tarsal: (0) present, (1) absent.
194) Second distal tarsal: (0) present, (1) absent.
195) Fifth distal tarsal: (0) present, (1) absent.
196) Metatarsal five, proximal, postaxial: (0) smooth, curved margin; (1) prominent pointed process (outer process sensu Robinson 1975) present.
197) Metatarsal five, distal shaft, angle relative to proximal tarsal articulation: (0) straight, with proximal tarsal articulation forming straight line with primary shaft; (1) "hooked," with proximal tarsal articulation forming right angle with primary shaft.
198) Metatarsal five, concavity along preaxial margin; (1) present, (1) absent, metatarsal five blocky in shape.
199) Pedal digit five, proximal phalanx: (0) shorter than proximal phalanx of digit four; (2) proximal phalanx elongate, longer than all other proximal phalanges.
200) Heterotopic ossifications: (0) absent in a minimum of 5 individuals, (1) present.
201) Maxilla, medial surface dorsal to tooth row: (0) smooth, (1) prominent anteroposteriorly oriented ridge present.
202) Maxilla, dorsal process, shape: (0) posteriorly concave margin, (1) simply tapers to point dorsally.
203) Maxilla, anterolateral surface: (0) large anteriorly opening foramen present, positioned just anterodorsal to primary row of neurovascular foramina; (1) foramen absent.
204) Maxilla, anteromedial surface, palatal process: (0) absent, (1) present, but fails to reach the midline; (2) present and touches its antimere at the midline. ORDERED.
    - This character was not ordered in the analysis of Pritchard and Nesbitt (2017), unlike the original usage in Nesbitt (2011). It has been ordered for this analysis.
205) Jugal, anterior process: (0) slender and tapering, (1) broad and expanded anteriorly.
206) Ectopterygoid, articulation with the pterygoid: (0) contacts part but not entirety of lateral edge of pterygoid, (1) contacts entire lateral edge of pterygoid.
207) Quadrate, proximal portion, posterior side: (0) continuous with the shaft, (1) expanded and hooked.
208) Parabasisphenoid, orientation of long axis: (0) horizontal, (1) more vertical.
209) Parabasisphenoid, semilunar depression on the lateral surface of the basal tubera: (0) present, (1) absent.
210) Dentary, posteroventral portion: (0) just meets the angular, (1) laterally overlaps the anteroventral portion of the angular (posteroventral process sensu Ezcurra, 2016[42]).
211) Dentition, crown height of the upper dentition compared with lower dentition: (0) similar tooth crown height, (1) upper dentition is shorter relative to taller lower dentition.
212) Antorbital fossa: (0) restricted to the lacrimal; (1) restricted to the lacrimal and dorsal process of the maxilla; (2) present on the lacrimal dorsal process of the maxilla and the

dorsal margin of the posterior process of the maxilla, the ventral border of the antorbital fenestra. ORDERED.

- This character was not ordered in the analysis of Pritchard and Nesbitt (2017), unlike the original usage in Nesbitt (2011). It has been ordered for this analysis.

213) Anterior cervical vertebrae (presacral vertebrae 3–5): (0) postzygapophyses separated posteriorly, (1) connected through a horizontal lamina (=transpostzygapophyseal lamina) with a notch at the midline.

214) **Cervical vertebrae ratio of lengths of fourth or fifth cervical centra to heights of anterior articular surfaces: (0) <1, (1) 1–3; (2) 3–10, (3) >10. ORDERED.**

- The character employed originally for Nesbitt (2011) only distinguished between vertebral centra that are equivalent in anteroposterior length and dorsoventral height and those with a greater anteroposterior length. We here follow an approach similar to [42], distinguishing between a wider range of relative lengths in Triassic archosauromorphs.

215) Dorsal vertebrae, diapophysis, anteroposteior position: (0) anterior portion of the neural arch and/or centrum, (1) anteroposterior middle of the neural arch and/or centrum.

216) Sacral ribs, anteroposterior length of first primordial sacral rib versus second primordial sacral rib: (0) longer anteroposteriorly than primordial sacral rib two, (1) about the same length or longer anteroposteriorly than second primordial sacral rib.

217) Anterior caudal vertebrae, neural spines: (0) inclined posteriorly, (1) vertical.

218) Caudal vertebrae, length of the anterior caudal vertebrae (caudal vertebrae 1–10) relative to posterior caudal vertebrae (~25): (0) nearly the same length, (1) posterior caudal vertebrae much longer.

219) Scapula, anterior margin: (0) straight or partially concave, (1) markedly concave.

220) Scapula, scapular blade, ratio of dorsoventral height to anteroposterior length at base of blade: (0) <.4, (1) .4–.25, (2) >.25. ORDERED.

221) Humerus, distal end, transverse width: (0) less than 2.5 times the minimum width of the shaft, (1) equal or more than 2.5 times the minimum width of the shaft.

222) Manual ungual, length: (0) about the same length or shorter than the penultimate phalanx of the same digit, (1) distinctly longer than penultimate phalanx of the same digit.

223) Ilium, acetabulum, ventral margin: (0) convex, (1) concave.

224) Ilium, iliac blade, maximum anteroposterior length: (0) less 3 times maximum dorsoventral height, (1) more than 3 times maximum dorsoventral height.

225) Ischium, anteroposterior length: (0) about same length or shorter than dorsal margin of iliac blade, (1) markedly longer than dorsal margin of iliac blade.

226) Femur, ridge of attachment of the *M. caudifemoralis*: (0) bladelike with a distinct asymmetric apex located medially (=internal trochanter), (1) low and without a distinct medial asymmetrical apex (=fourth trochanter).

227) Femur, anterior trochanter (*M. iliofemoralis cranialis* insertion): (0) absent, (1) present.

228) Astragalus, tibial and fibular articulations: (0) separated by a gap or notch of Gower 1996, (1) continuous.

229) Calcaneum calcaneal tuber (=primordial lateral projection in early diapsid groups), shaft proportions at the midshaft of the tuber: (0) taller than broad, (1) about the same or broader than tall.

230) Calcaneum, articular surfaces for fibula and distal tarsal IV: (0) separated by a nonarticular surface, (1) continuous.

231) Calcaneum, tuber (=primordial lateral projection in early diapsid groups), orientation relative to the transverse plane: (0) lateral, less the 20 degrees; (1) posteriorly deflected, between 21 49 degrees; (2) posterolaterally between 50 90 degrees posteriorly. ORDERED.
232) Metatarsal IV, proximodistal length: (0) longer than metatarsal III, (1) about the same length or shorter than metatarsal III.
233) Pes, unguals, ventral tubercle: (0) absent or small, (1) well developed and extended ventral to proximal articular facet of ungual.
234) Distal non-ungual pedal phalanges, distal articular portion: (0) lateral and medial sides parallel or near parallel, (1) lateral and medial sides converging anterodorsally.
235) Pes, penultimate phalanx, proximodistal length: (0) shorter than the more proximal phalanx, (1) significantly longer than the more proximal phalanx.
236) Osteoderms: (0) absent, (1) present.
237) Prefrontal, orbital margin: (0) lateral surface smooth or with slight grooves; (1) rugose lateral sculpturing present.
238) Gastralia: (0) abundant, with individual gastral elements nearly contacting medially; (1) small in number, well separated, or unossified.
239) Astragalus, margin between tibial and fibular facets: (0) grades smoothly into anterior hollow of astragalus, (1) prominent ridge separates margin from anterior hollow.
240) Proximal tarsals, morphology of perforating foramen: (0) broad, marked by finished bone on astragalus and calcaneum; (1) pinched, marked by extremely constricted space between astragalus and calcaneum.
241) Dentary, anterior portion: (0) in same horizontal plane as anteroposterior middle portion of dentary, (1) anteroventrally deflected relative to anteroposterior middle portion of dentary.
242) Quadrate, posterior margin, ventral half: (0) flat or slightly concave, (1) distinctly convex.
243) Atlas, centrum: (0) separate from axial intercentrum, (1) fused to axial intercentrum.
244) Axis, neural spine: (0) dorsal margin inclined anteroventrally, (1) dorsal margin inclined anterodorsally.
245) Presacral vertebrae (5th vertebra to the sacrum), neural arch, posterior edge: (0) spinopostzygapophyseal laminae absent, (1) spinopostzygapophyseal laminae present.
246) Dentary, lateral exposure, posterior extent: (0) posteriormost extent of dentary on dorsum of mandible -posterodorsal process of dentary sensu [42]]; (1) posteriormost extent of dentary positioned ventral to surangular (posterocentral process of dentary sensu [42]).
247) Premaxilla, medial surface, contribution to palate: (0) absent, (1) flattened palatal process present.
248) Premaxilla, palatal process: (0) extends posteriorly as far as the lateral surface of the premaxilla, (1) extends posteriorly past the lateral surface of the premaxilla.
249) Premaxilla, fusion to contralateral premaxilla: (0) absent, (1) present;
250) Premaxilla, tooth morphology: (0) similar in morphology to maxillary teeth, (1) single, apicobasally elongate, chisel-like tooth, (2) teeth longer than maxillary teeth with subcircular cross section.
251) Maxilla, dentigerous surface, ventral surface: (0) ungrooved; (1) single, anteroposteriorly running groove; (2) two anteroposteriorly running grooves. ORDERED.

252) Nasals, anterior margins: (0) appressed nasals form anteriorly pointed structure in midline, (1) appressed nasals form anteriorly flattened surface, (2) appressed nasals are separated anteriorly by premaxilla.
253) Lacrimal: (0) present as distinct ossification, (1) absent.
254) Postfrontal, shape of dorsal exposure: (0) forms a right triangle with right angle forming posteromedial margin, (1) anteroposteriorly broad, posterior margin inclined posteromedially.
255) Parietal, posterolateral (=post-temporal) process: (0) slender and tapering; (1) anteroposteriorly flattened, such that parietal contributes prominently to occipital face of skull.
256) Parietal, posterolateral (=post-temporal) process: (0) lateral ornamentation absent, margin is smooth; (1) ornamentation present, margin marked by dorsolaterally oriented row of pointed projections.
257) Skull roof, upper temporal fenestra: (0) absent, dorsal skull roofing bones cover adductor chamber; (1) present as gap between dorsal exposures of skull roofing bones.
258) Skull roof, posterolateral surface, ornamentation: (0) absent, (1) prominent horns on squamosal and quadratojugal.
259) Quadrate, pterygoid ramus, ventral margin of posterior base of ramus: (0) in line with quadrate condylar surface, (1) elevated dorsally relative to quadrate condylar surface.
260) Suborbital fenestra: (0) absent, no gap between palatine ectopterygoid and pterygoid; (1) present, cavity on the palate present between palatine ectopterygoid and pterygoid.
261) Palatine, posterolateral portion, transverse expansion: (0) absent, producing anteriorly curved suborbital fenestra; (1) present, producing anteriorly tapered suborbital fenestrae.
262) Prootic, foramen for facial nerve, lateral exit: (0) sits within broad open surface along anterior inferior process, (1) exit sandwiched between crista prootica anterodorsally and an additional thin plate of bone posteroventrally.
263) Parabasisphenoid, cultriform process, dentition: (0) teeth run anteroposteriorly on process, (1) teeth clustered at base of process.
264) Stapes, stapedial shaft, robusticity: (0) robust with thick shaft, similar or greater in breadth to the paroccipital process of the opisthotic; (1) slender with rod-like shaft, much slenderer than paroccipital process.
265) Surangular, dorsolateral surface: (0) transversely narrow, (1) exhibits transversely wide shelf.
266) Articular, fossa posterior to glenoid: (0) subequal or greater in anteroposterior length to the glenoid fossa; (1) anteroposteriorly constricted, shorter than the glenoid fossa.
267) Articular, retroarticular process, dorsoventral dimensions: (0) shallow, dorsal margin positioned posteroventral to quadrate articulation; (1) deep, dorsal margin at dorsoventral level equivalent to quadrate articulation.
268) Articular, retroarticular process, posterior tip: (0) oriented posteriorly oriented, (1) posterodorsally upturned.
269) Axis intercentrum, fusion to axial centrum: (0) absent, (1) present.
270) Post axial cervical vertebrae, intervertebral articulations: (0) circular or ovoid articular surfaces appressed to one another directly, (1) saddle-shaped articular surface (=heterocoely).
271) Anterior cervical vertebra, postzygapophysis, epipophysis, morphology: (0) form vertical expansions of bone above facet but do not extend posteriorly beyond facet, (1) form

posteriorly pointed projections that barely project posteriorly beyond the level of the facet, (2) form posteriorly pointed projections that project far posteriorly beyond the level of the facets. ORDERED.

272) Anterior cervical vertebra, hypapophysis: (0) absent, ventral surface of centrum unexpanded, (1) posteroventrally posteroventral surface of centrum exhibits massive posteroventrally projecting crest.

273) Cervical ribs: (0) present as distinct ossifications from cervical vertebrae, (1) absent as distinct ossifications.

274) Anterior dorsal vertebra, neural arch, surface ventrolateral to base of neural spine: (0) smooth, (1) surface marked by deep concavity.

275) Anterior dorsal vertebra, pedicel, dorsoventral height: (0) substantially shorter than respective centra, (1) taller than respective centra.

276) Anterior dorsal vertebra, neural spine, anteroposterior expansion: (0) remain roughly similar in anteroposterior length throughout dorsoventral height; (1) dorsally broader anteroposteriorly than at spine base; (2) third dorsal spine anteroposteriorly expanded into hatchet shape, contacting other dorsal neural spines. ORDERED.

277) Dorsal ribs, accessory ossification at distal tip: (0) absent, (1) present.

278) Mid-dorsal ribs, fusion to respective centra: (0) absent, (1) present.

279) Dorsal ribs, curvature of rib shaft: (0) curve ventromedially to frame trunk; (1) splayed laterally, forming patagium.

280) Anterior caudal vertebra, neural spine, dorsoventral height: (0) similar in height or shorter than sacral neural spines, (1) taller than sacral neural spines.

281) Caudal vertebra, anterior neural spine, anteroposterior expansion of spine tip: (0) unexpanded dorsally, (1) slender anterior and posterior projections forming T shape.

282) Chevron, proximal articular morphology: (0) unfused to centra, (1) fused to centra.

283) Anterior chevron, hemal spine, morphology: (0) forms single spine, (1) bifurcates ventrally.

284) Anteriormost chevron, hemal spine, morphology: (0) bifid spines remain separate ventrally, (1) bifid spines recontact ventrally forming foramen.

285) Posterior chevron, proximal articulation: (0) positioned intervertebrally, (1) positioned at anteroventral surface of centrum.

286) Terminal caudal vertebra(e): (0) similar in morphology to other posterior caudals, (1) modified into claw-like element.

287) Supraneural ossification (bone growth positioned anterodorsal to anterior dorsal neural spines): (0) absent, (1) present.

288) Scapulocoracoid, glenoid fossa, position: (0) at or near base of scapular blade, (1) located far ventral of base of scapular blade.

289) Scapulocoracoid, glenoid fossa, orientation relative to long axis of trunk: (0) oriented posterolaterally, ventral margin extends posterior of dorsal margin; (1) oriented laterally, ventral margin positioned directly underneath to dorsal margin.

290) Humerus, internal tuberosity: (0) continuous with humeral shaft, (1) offset from humeral shaft by cylindrical pedicel of finished bone.

291) Humerus, epicondyles, proximal base: (0) positioned distal to midshaft, (1) positioned at near midshaft.

292) Humerus, ectepicondyle, morphology of lateral margin: (0) squared off pre axially, (1) pointed triangular pre axially.

293) Humerus, entepicondyle, distal extent: (0) terminates proximal to the distal margin of the ulnar condyle, (1) extends distally relative to ulnar condyle.

- Taxa coded as “0” for character 154 are coded as “-“ for this character.

294) Humerus, distalmost end: (0) collinear with proximal shaft, (1) primary axis of shaft curves towards flexor surface at distal end.

295) Radius, proximal tab: (0) absent, (1) prominent tab for articulation with ulna present.

296) Radius, distal articulation: (0) terminal surface is concave, (1) small styloid process on radius fits into radiale.

297) Ulna, shape: (0) similar to radius with elongate shaft, (1) flattened in pre-axial/postaxial plane forming enormous crescent.

298) Ulnare and intermedium, proximodistal elongation: (0) longer proximodistally than in pre axial post axial plane; (1) elements short, equivalent in proximodistal and pre axial post axial length.

299) Second manual ungual: (0) similar in morphology to other manual, (1) substantially taller and more massive than other manual unguals.

300) Manual digit three III, phalangeal formula: (0) multiple phalanges, (1) single non ungual phalanx.

301) Ilium, anteroventral margin of iliac blade: (0) inclined posterodorsally, (1) inclined vertically, (2) inclined anterodorsally. ORDERED.

302) Ilium, iliac blade, post-acetabular portion: (0) relatively planar or lightly sculptured; (1) marked by posterodorsally running ridge, extending from posterior margin of supraacetabular margin.

303) Femur, proximal surface, dorsal surface: (0) unornamented, (1) marked by prominent tuberosity.

304) Calcaneum, primordial lateral projection, ventral margin: (0) convex and continuous with the lateral margin of the projection; (1) margin is concave, sharply angled relative to lateral margin of the projection.

305) Metatarsal I, shaft, proximodistal length relative to proximodistal length of metatarsal IV: (0) >0.42, (1) 0.42–0.32, (2) <0.32. ORDERED.

306) Pes digit three III, phalangeal formula: (0) four, (1) three.

307) Anterior cervical vertebra, neural arch, transverse breadth at anteroposterior midpoint relative to centrum: (0) subequal, (1) substantially broader.

308) Rostrum, anteroposterior length relative to total anteroposterior length of skull: (0) <0.40; (1) 0.41–0.62; (2) >0.62. ORDERED.

- Character modified from[40,42,103]. Following character 20 of [42], we divide the relative rostrum lengths into a series of discretized states. Contra [42], we do not incorporate taxa with snout lengths greater than 70% the total length of the skull (e.g., Phytosauria), and we thus incorporate a lower bound for the third state in this character.

309) Premaxilla, posterodorsal process, posterior surface: (0) marked by prominent, transversely compressed lamina linking posterior tip of process to alveolar portion of bone; (1) lamina absent.

- NOVEL CHARACTER. This character describes a curious feature of the posterodorsal process of the premaxilla in *Clevosaurus*, based primarily on observations of the isolated bone in *C. hudsoni* (e.g., NHMUK R 676-677). In those animals, the anterior surface of the maxilla is underlain by a transversely

narrow lamina of the premaxilla. This feature is illustrated in Fig. 5 of [80]. It also occurs in the Indian rhynchocephalian *Godavarisaurus lateefi*[104].

- In most archosauromorphs, the posterodorsal process of the premaxilla is flat on its posterior surface (e.g., the pan-archosaurs *Prolacerta broomi*, BP/1 2975; *Trilophosaurus buettneri*, TMM 31025-207), or possesses a slight posterior tuberosity (e.g., the pan-archosaur *Azendohsaurus madagaskarensis*, FMNH PR 2751). In rhynchosaurs, the maxilla laps laterally over the posterodorsal process of the premaxilla (e.g., the rhynchosaur *Mesosuchus browni*, SAM-PK 6536; *Rhynchosaurus articeps*, NHMUK R1237); however, there is no similar posterior lamina in the premaxillae of those taxa.

310) Maxilla, dorsal process, anterior surface: (0) posterodorsally inclined, (1) dorsally inclined.

- NOVEL CHARACTER. In most diapsids that possess a dorsal process of the maxilla, the anterior surface of the bone is inclined posterodorsally. In some rhynchocephalians (e.g., *Clevosaurus hudsoni*[80] *Clevosaurus brasiliensis*[79]), the anterior surface of the maxilla is extremely steeply inclined, nearly ninety degrees relative to the long axis of the bone.

311) Nasal, lateral surface: (0) meets dorsoventrally short length of medial surface of dorsal process/portion of the maxilla, (1) meets entire dorsoventral height of medial surface of supra-alveolar portion of maxilla.

- NOVEL CHARACTER. In nearly all taxa integrated into this analysis, the nasal meets the dorsal margin of the maxilla along some portion of the anteroposterior length of the rostrum. In most diapsid reptiles, this contact is dorsoventrally short, occupying a small amount of the dorsoventral height of the supra-alveolar portion of the maxilla (e.g., the pan-archosaurs *Prolacerta broomi*, SAM-PK 10018, UCMP 37151; *Batrachotomus kupferzellensis*[57]; the squamate *Iguana iguana*, YPM R 13952). State 0 is illustrated by a specimen in *Prolacerta broomi* that is missing the anterodorsal portion of its left maxilla in Fig. S12A.
- In several diapsid lineages, the nasal has a proportionally taller lamina that fits against most or all of the supra-alveolar portion of the maxilla. This tall lamina is present in *Sphenodon punctatus*[97], a number of rhynchosaurs (e.g., *Mesosuchus browni*, SAM-PK 6536; *Teyumbaita sulcognathus*[52] and *Colobops noviportensis* (YPM VPPU 18835). This condition is illustrated using CT slice data in Fig. S11B and three-dimensional volume renderings in Fig. S12B.

312) Jugal, lateral surface, anteroposteriorly oriented shelf (i.e., *anguli oris* crest): (0) restricted to main body of bone, (1) extends at least to anterior tip of anterior process of bone.

- Modified from [42,105].
- In both [42,105], this character incorporates a state for taxa in which the lateral shelf (*anguli oris* crest) extends onto the lateral surface of the maxilla as well as the anterior process of the jugal.

313) Postfrontal, posteromedial corner: (0) anteroposteriorly or posterolaterally inclined, (1) posteromedially inclined, resulting in transverse tapering of posterior portion of frontal.

- NOVEL CHARACTER.
- In most Permian and Triassic Diapsida, the contact between the frontal and postfrontal is either oriented in a parasagittal plane or inclined posterolaterally,

owing to transverse expansion of the former bone. Such conditions occur in the non-saurian diapsid *Youngina capensis* (e.g., BP/1 3859), the pan-archosaur *Protorosaurus speneri*[43], *Proterosuchus fergusi* (SAM-PK 10603), and the rhynchocephalian *Clevosaurus hudsoni* (e.g., NHMUK R 36832).
- In a number of early archosauromorph taxa, including Rhynchosauria, the postfrontal encroaches medially at the level of the frontoparietal suture. As a consequence, the frontals taper medially at their posterior tips. This condition can be seen in the pan-archosaurs *Mesosuchus browni* (SAM-PK 6536), *Hyperodapedon sanjuanensis* (MCZ 1636), *Trilophosaurus buettneri* (TMM 31025-140), and *Azendohsaurus madagaskarensis* (UA-7-20-99-653).

314) Postorbital, medial process, contact with postfrontal: (0) positioned posteroventral relative to postfrontal, (1) medial process laps dorsally over postfrontal.
- Modified from [106].

315) Upper temporal bar (contributions of postorbital and squamosal): (0) located approximately at dorsoventral midpoint of orbit, (1) located approximately aligned to dorsal border of orbit.
- Equivalent to [42].
- We illustrate our interpretations of these states and our reconstruction of the position of the bar relative to the orbit in *Colobops noviportensis* in Fig. S13.

316) Quadrate, dorsalmost portion: (0) tapering dorsally, (1) dorsally expanded into prominent convexity (cephalic condyle *sensu* [107]).
- Modified from [108].
- The term "cephalic condyle" is most often applied to the dorsal convexity on the quadrates of lepidosauromorph reptiles[88,107,109]. We here consider the similar convexity present in a wide range of early pan-archosaurs (e.g., *Prolacerta broomi*, BP/1 5375; *Mesosuchus browni*, SAM-PK 6536; *Azendohsaurus madagaskarensis*, FMNH PR 2751) to be homologous, such that the term would be applicable to both lepidosauromorphs and archosauromorphs.

317) Ectopterygoid, posterior expansion of lateral process in contact with jugal: (0) absent; (1) present, with prominent posterior process extending posteriorly from primary axis of lateral process.
- Equivalent to [42].

318) Basioccipital, ventral margin: (0) prominent embayment between basal tubera at least as transversely broad as occipital condyle, (1) transversely narrow embayment between basal tubera, narrower than occipital condyle.
- NOVEL CHARACTER.
- In most early Diapsida and many early Sauria, the ventral margin of the basioccipital exhibits a deep, ventrally open concavity that is transversely broader than the transverse width of the occipital condyle. This condition occurs in *Avicranium renestoi* (Pritchard and Nesbitt, 2017), *Youngina capensis* (Gardner et al., 2010), *Clevosaurus hudsoni* (NHMUK PLR 606), and *Tanystropheus longobardicus*[46].
- In many derived archosauromorphs, there is a much narrower ventral embayment between the basal tubera of the basioccipital. In these taxa, the embayment is transversely much narrower than the transverse width of the occipital condyle. This condition occurs in *Mesosuchus browni* (SAM-PK 6536), *Trilophosaurus*

buettneri (TMM 31025-244), *Kuehneosaurus latus* (NHMUK R 6059), *Azendohsaurus madagaskarensis* (FMNH PR 2765), and *Proterosuchus alexanderi* (NMQR 1484).

319) Mandible, coronoid process, dorsoventral height: (0) dorsoventrally shorter than anterior process of jugal; (1) dorsoventrally tall, equivalent or taller than anterior process of jugal.
- Coronoid processes are actually quite common among early Archosauromorpha, although their relative level of development is quite distinct. In some tanystropheids [e.g., *Macrocnemus bassanii* (PIMUZ T/2472), *Tanystropheus longobardicus* (MCSN BES SC 1018)], the process is dorsoventrally short and anteroposteriorly narrow, barely extending into the temporal cavity.
- In contrasts, the coronoid processes in some other archosauromorphs [e.g., *Trilophosaurus buettneri* (TMM 31025-140), *Langobardisaurus pandolfii* (MFSN 1921), and *Hyperodapedon sanjuanensis* (MCZ 1636)], lepidosaurs [e.g., *Clevosaurus hudsoni* (NHMUK R 36832), *Uromastyx acanthinura* (YPM R 13525)], and *Colobops noviportensis* (YPM VPPU 18835), which is proportionally much taller. In these taxa, the process is higher than the anterior process of the jugal, extending well into the temporal region.

320) Dentary, contribution to lateral mandibular surface: (0) terminates at or near posterior terminus of tooth row, (1) extends posteriorly to overlap coronoid, (2) extends well posterior to coronoid. ORDERED.
- Modified from [110] and [106]. Incorporates states similar to reference [42].

321) Splenial: (0) present as distinct ossification, (1) absent as distinct ossification.
- Modified from references [110] and [106].

322) Dorsal vertebra, neural spine, dorsal expansion (spine table): (0) little lateral expansion relative to base of neural spine; (1) transversely broad, wider than base of neural spine.
- Equivalent to [49]. This character was not presented in the analysis of reference [100] but it is included here to account for variation seen in Tanystropheidae.

323) Metatarsal five, ratio of proximodistal length of contact with distal tarsal four to proximodistal length of distal process: (0) >1, (1) <1.
- NOVEL CHARACTER.
- In most diapsid reptiles, the length of the contact between the fourth distal tarsal and the fifth metatarsal is equivalent to or shorter than the proximodistal length of the main shaft of the bone distal to that contact. This is the case in *Youngina capensis*[21], *Clevosaurus hudsoni* (NHMUK PLR 600a), *Prolacerta broomi* (BP/1 2676), *Trilophosaurus buettneri* (TMM 31025-140), and *Macrocnemus bassanii* (PIMUZ T/4822).
- In a number of tanystropheids, the distal portion of the fifth metatarsal is shorter than the contact surface for the fourth distal tarsal. This condition occurs in *Amotosaurus rotfeldensis* (SMNS 54783), *Tanystropheus longobardicus* (MCSN BES SC 1018), and *Langobardisaurus pandolfii* (MFSN 1921).

Supplementary Note 4: Modifications to characters and codings from the matrix of Pritchard and Nesbitt (2017)

The following are codings changed from the dataset of ref [100]. Some are corrections from the original dataset, whereas others are revised based on firsthand studies of material that was not

incorporated into ref [100]. Changes are only included for characters that were unchanged from the original study or changed only slightly (indicated in **bold** above).

7)
1->0
Clevosaurus hudsoni

9)
?->1
Diphydontosaurus avonis

10)
?->0
Kuehneosaurus latus

16)
?->1
Diphydontosaurus avonis

20)
?->0.
Diphydontosaurus avonis
0->1
Gephyrosaurus bridensis

24)
?->0
Clevosaurus hudsoni

25)
0->?
Gephyrosaurus bridensis

28)
?->0
Kuehneosaurus latus

33)
1->0
Diphydontosaurus avonis

34)
?->1.
Diphydontosaurus avonis

36)

?->0
Diphydontosaurus avonis

37)
?->0
Kuehneosaurus latus

40)
0->?
Gephyrosaurus bridensis

41)
?->1
Pamelaria dolichotrachela

43)
?->0
Pamelaria dolichotrachela
?->1
Diphydontosaurus avonis
0->1
Clevosaurus hudsoni

45)
?->0
Clevosaurus hudsoni
Kuehneosaurus latus

46)
?->0
Kuehneosaurus latus

50)
?->1
Clevosaurus hudsoni

51)
0->1
Kuehneosaurus latus

52)
?->1
Kuehneosaurus latus

55)
?->1

Kuehneosaurus latus

56)
?->0
Kuehneosaurus latus

68)
1->1+2
Kuehneosaurus latus

70)
0->1
Kuehneosaurus latus

74)
?->0
Kuehneosaurus latus

79)
?->0
Kuehneosaurus latus

81)
?->0.
Diphydontosaurus avonis

82)
?->1
Gephyrosaurus bridensis

83)
?->1
Gephyrosaurus bridensis
Pamelaria dolichotrachela
0->1
Kuehneosaurus latus

86)
?->0
Planocephalosaurus robinsonae

87)
?->1
Planocephalosaurus robinsonae

88)

?->0
Planocephalosaurus robinsonae

104)
?->0
Clevosaurus hudsoni
Kuehneosaurus latus

105)
?->1
Kuehneosaurus latus

107)
?->0
Diphydontosaurus avonis

117)
?->0
Diphydontosaurus avonis

119)
?->0
Diphydontosaurus avonis

122)
1->2
Kuehneosaurus latus

124)
?->1
Kuehneosaurus latus

125)
?->0
Diphydontosaurus avonis
?->1
Tanytrachelos ahynis

126)
?->1
Diphydontosaurus avonis
Tanytrachelos ahynis

131)
?->1
Diphydontosaurus avonis

0->1
Kuehneosaurus latus

136)
?->1
Kuehneosaurus latus

139)
?->1
Diphydontosaurus avonis
0-?
Clevosaurus hudsoni

140)
?->1
Clevosaurus hudsoni

141)
?->1
Clevosaurus hudsoni

142)
?->1
Kuehneosaurus latus

143)
?->0
Clevosaurus hudsoni
Kuehneosaurus latus

144)
?->0
Kuehneosaurus latus

148)
0->1
Pamelaria dolichotrachela

149)
?->0
Clevosaurus hudsoni

?->1
Clevosaurus hudsoni

158)

?->1
Clevosaurus hudsoni
Diphydontosaurus avonis

159)
?->1
Clevosaurus hudsoni
Diphydontosaurus avonis

160)
?->0
Clevosaurus hudsoni
Tanytrachelos ahynis

161)
?->1
Clevosaurus hudsoni

162)
?->0
Clevosaurus hudsoni

163)
1->0+1
Kuehneosaurus latus

165)
0->1
Kuehneosaurus latus

169)
0->1
Kuehneosaurus latus

170)
?->0
Kuehneosaurus latus
0->1
Pamelaria dolichotrachela

173)
?->1
Pamelaria dolichotrachela

174)
?->0

Pamelaria dolichotrachela

175)
?->1
Kuehneosaurus latus
Pamelaria dolichotrachela

178)
0->1
Kuehneosaurus latus
1->0
Clevosaurus hudsoni

179)
1->0
Clevosaurus hudsoni

181)
?->1
Diphydontosaurus avonis

182)
?->1
Clevosaurus hudsoni

184)
?->0
Kuehneosaurus latus
1->0
Diphydontosaurus avonis

185)
1->0
Clevosaurus hudsoni

186)
?->0
Kuehneosaurus latus
?->1
Diphydontosaurus avonis

187)
?->0
Diphydontosaurus avonis

188)

?->0
Diphydontosaurus avonis

189)
?->0
Diphydontosaurus avonis

?->0
Diphydontosaurus avonis

192)
?->1
Diphydontosaurus avonis

196)
?->1
Kuehneosaurus latus

197)
0->1
Kuehneosaurus latus

198)
?->0
Kuehneosaurus latus

199)
?->0
Diphydontosaurus avonis

202)
?->1
Kuehneosaurus latus

203)
?->0
Kuehneosaurus latus

206)
?->0
Kuehneosaurus latus
?->1
Planocephalosaurus robinsonae

207)
?->0

Clevosaurus hudsoni

209)
?->0
Clevosaurus hudsoni

210)
?->0
Kuehneosaurus latus

211)
?->0
Kuehneosaurus latus

215)
?->0
Diphydontosaurus avonis

217)
?->1
Kuehneosaurus latus

220)
0->1
Clevosaurus hudsoni

222)
?->0
Clevosaurus hudsoni
Tanytrachelos ahynis

227)
?->0
Diphydontosaurus avonis

228)
?->0
Diphydontosaurus avonis
Kuehneosaurus latus

229)
?->0
Clevosaurus hudsoni
Diphydontosaurus avonis

230)

?->0
Diphydontosaurus avonis

231)
?->0
Diphydontosaurus avonis

233)
?->0
Clevosaurus hudsoni

235)
?->0
Clevosaurus hudsoni

237)
?->0
Gephyrosaurus bridensis

238)
?->1
Kuehneosaurus latus
1->0
Diphydontosaurus avonis

239)
?->0
Diphydontosaurus avonis

240)
?->1
Kuehneosaurus latus

242)
?->1
Pamelaria dolichotrachela

243)
?->0
Kuehneosaurus latus
?->1
Clevosaurus hudsoni

244)
?->1
Clevosaurus hudsoni

245)
?->1
Kuehneosaurus latus

247)
1->0
Clevosaurus hudsoni

248)
1->-
Clevosaurus hudsoni

252)
?->0
Clevosaurus hudsoni
Diphydontosaurus avonis
?->1
Kuehneosaurus latus
?->2
Gephyrosaurus bridensis

256)
?->0
Diphydontosaurus avonis

262)
?->0
Kuehneosaurus latus

265)
?->0
Clevosaurus hudsoni
Planocephalosaurus robinsonae

266)
?->0
Planocephalosaurus robinsonae

267)
?->1
Planocephalosaurus robinsonae

268)
?->0
Planocephalosaurus robinsonae

269)
?->1
Clevosaurus hudsoni

270)
?->0
Diphydontosaurus avonis

272)
?->0
Diphydontosaurus avonis

273)
?->0
Diphydontosaurus avonis

274)
?->0
Diphydontosaurus avonis

275)
?->0
Diphydontosaurus avonis

276)
?->0
Diphydontosaurus avonis

277)
?->0
Gephyrosaurus bridensis

281)
?->0
Kuehneosaurus latus

282)
?->0
Kuehneosaurus latus

283)
?->0
Kuehneosaurus latus

285)

?->0
Kuehneosaurus latus

287)
?->0
Clevosaurus hudsoni
Diphydontosaurus avonis

289)
?->0
Clevosaurus hudsoni

292)
?->0
Clevosaurus hudsoni

295)
?->0
Kuehneosaurus latus
Tanytrachelos ahynis

296)
?->0
Clevosaurus hudsoni

297)
?->0
Kuehneosaurus latus

298)
?->0
Tanytrachelos ahynis
?->1
Clevosaurus hudsoni
Diphydontosaurus avonis

299)
?->0
Clevosaurus hudsoni
Tanytrachelos ahynis

300)
?->0
Clevosaurus hudsoni

301)

?->0
Diphydontosaurus avonis

303)
?->0
Clevosaurus hudsoni
Diphydontosaurus avonis

304)
?->0
Diphydontosaurus avonis

305)
?->0
Clevosaurus hudsoni

306)
?->0
Diphydontosaurus avonis

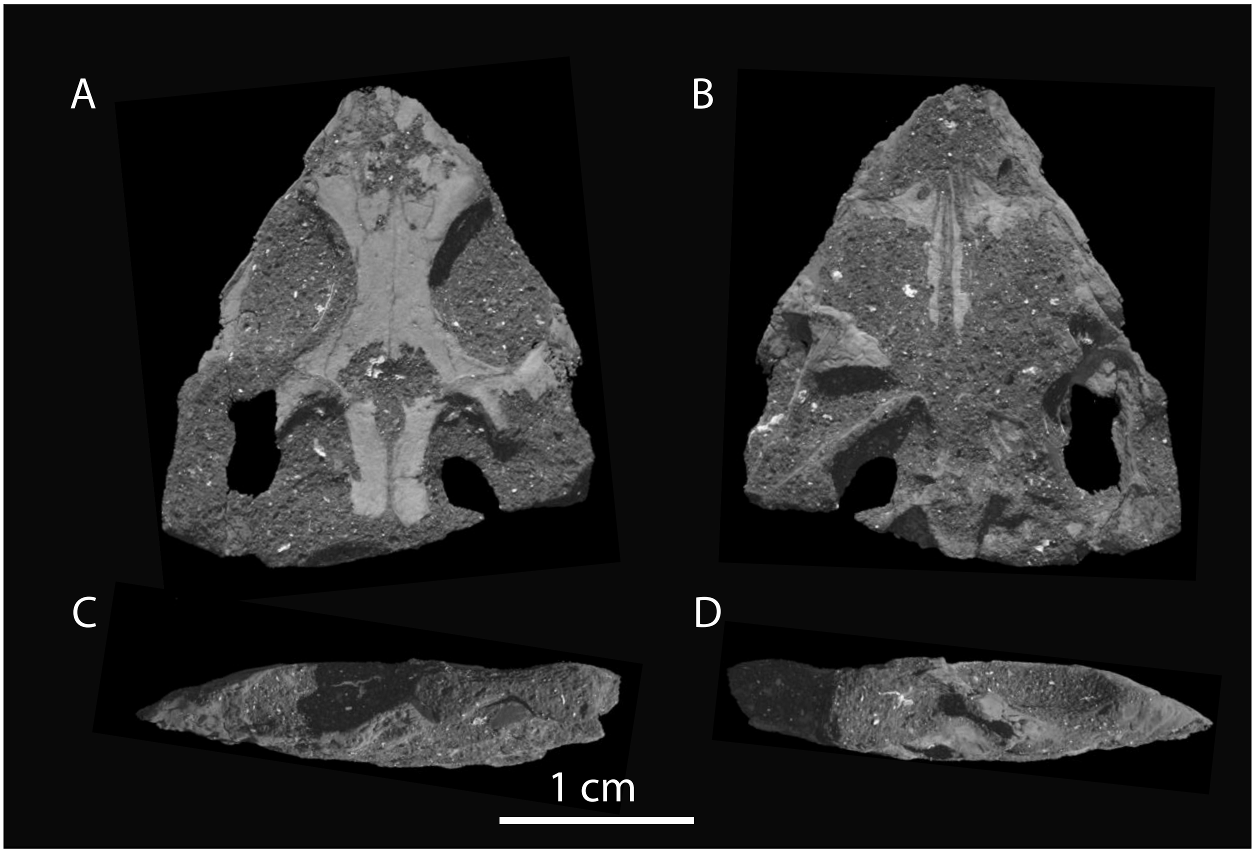

Supplementary Fig. 1. Volume rendering of the skull of *Colobops noviportensis* (YPM VPPU 18835) prior to three-dimensional segmentation and removal of matrix.

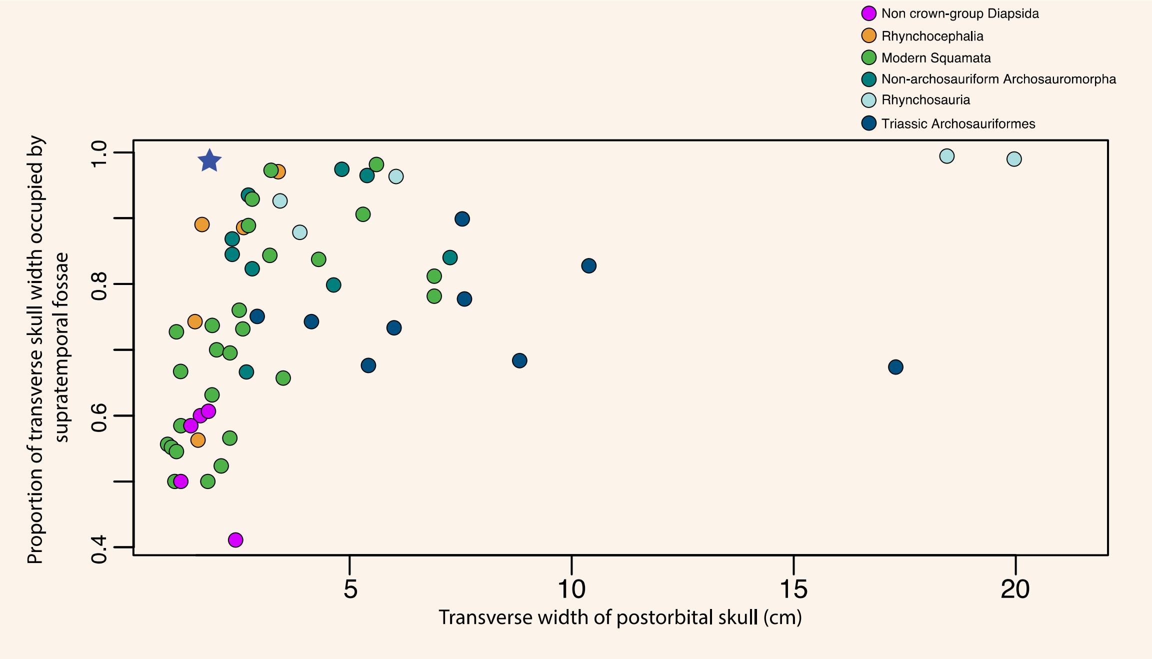

Supplementary Fig. 2. Bivariate plot of the transverse width of the postorbital portion of the skull (measured at the anteroposterior midpoints of the supratemporal fenestrae) against the proportional contribution of the supratemporal fossae to the transverse width of the postorbital portion of the skull. In contrast to the plot presented in Figure 3 in the main text, the x-axis is not log-scaled in this figure. The star symbol represents the position of *Colobops noviportensis* in morphospace.

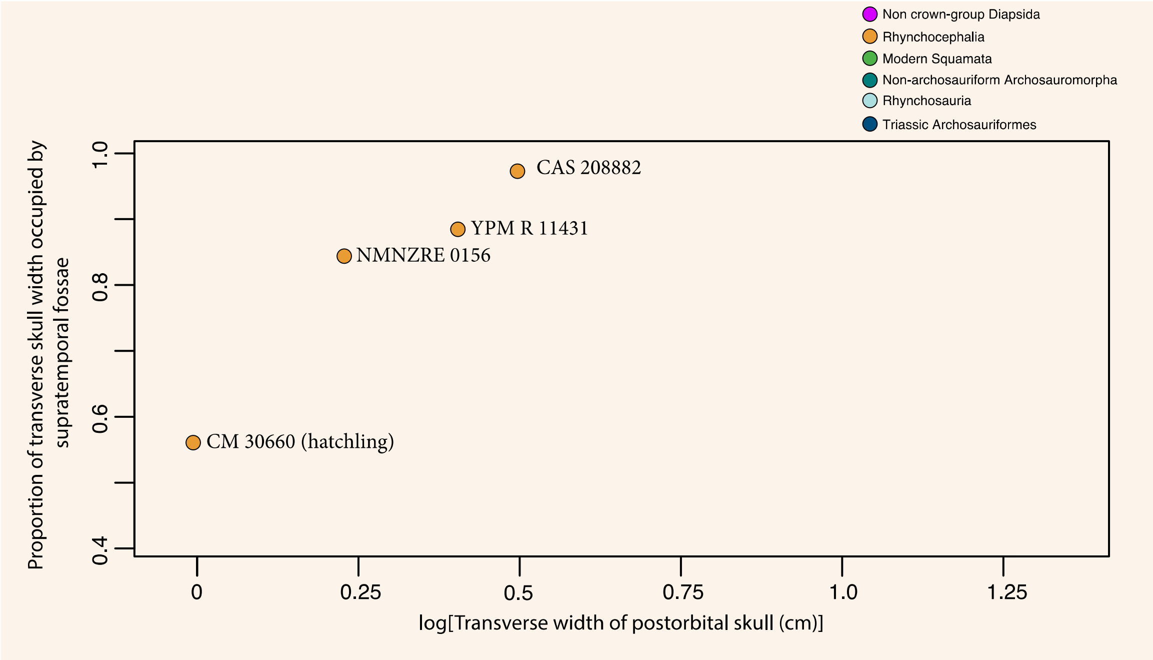

Supplementary Fig. 3. Bivariate plot of the transverse width of the postorbital portion of the skull (measured at the anteroposterior midpoints of the supratemporal fenestrae, log-scaled) against the proportional contribution of the supratemporal fossae to the transverse width of the postorbital portion of the skull. Plot illustrates *Sphenodon punctatus* specimens sampled.

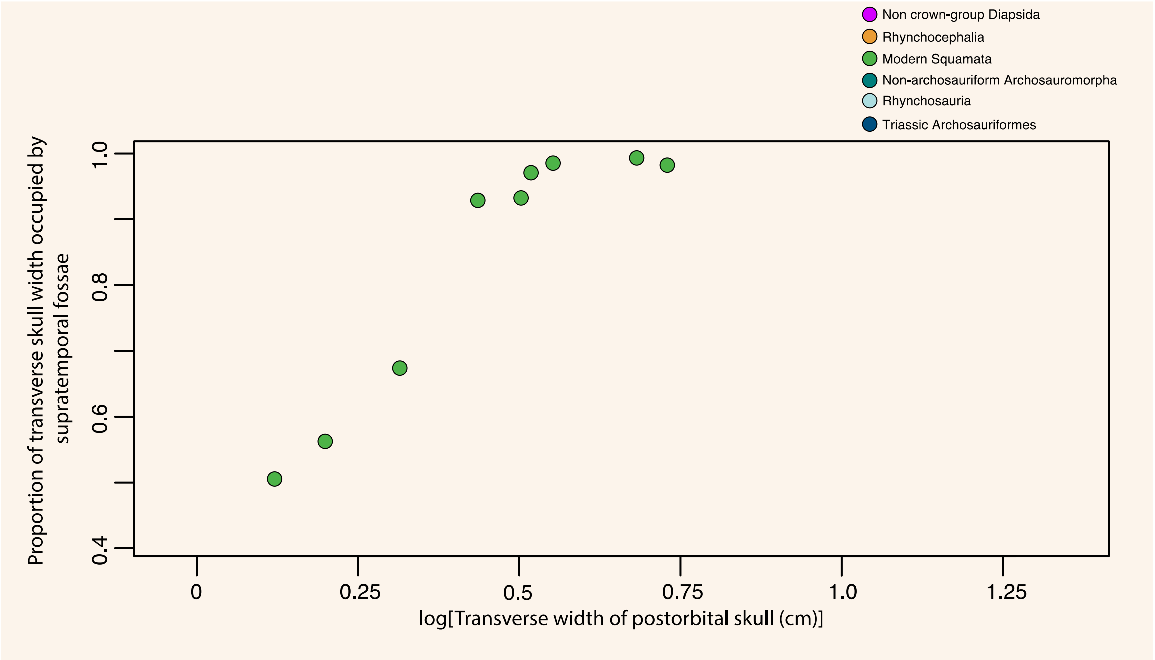

Supplementary Fig. 4. Bivariate plot of the transverse width of the postorbital portion of the skull (measured at the anteroposterior midpoints of the supratemporal fenestrae, log-scaled) against the proportional contribution of the supratemporal fossae to the transverse width of the postorbital portion of the skull. Plot illustrates *Iguana iguana* specimens sampled.

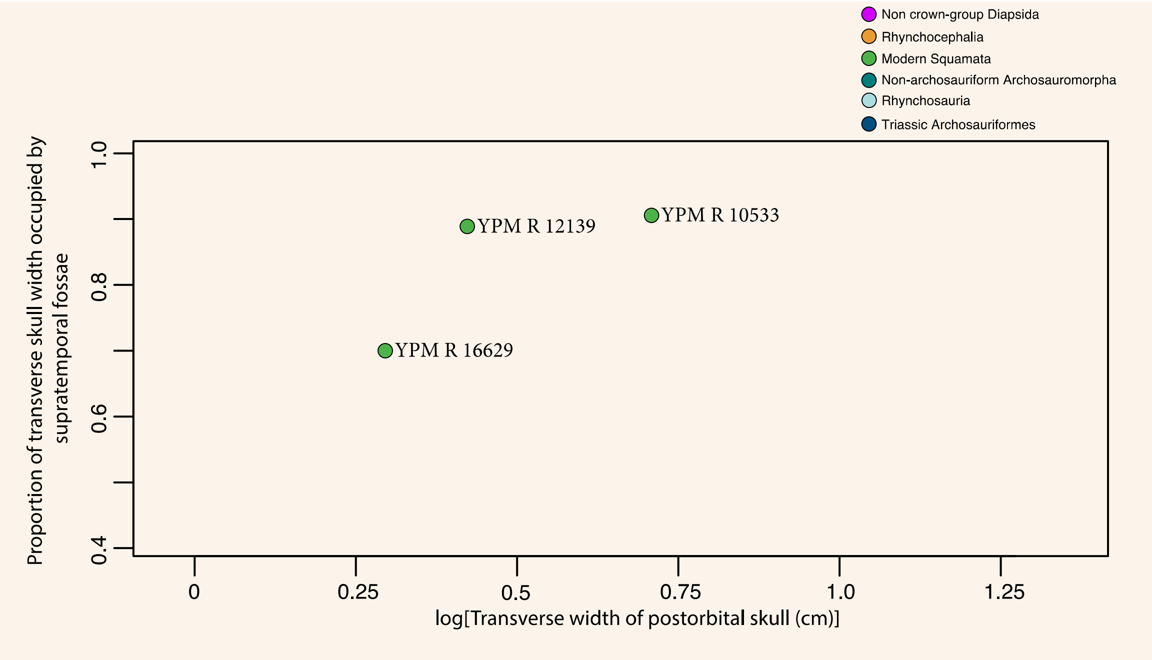

Supplementary Fig. 5. Bivariate plot of the transverse width of the postorbital portion of the skull (measured at the anteroposterior midpoints of the supratemporal fenestrae) against the proportional contribution of the supratemporal fossae to the transverse width of the postorbital portion of the skull. Plot illustrates *Pogona vitticeps* specimens sampled.

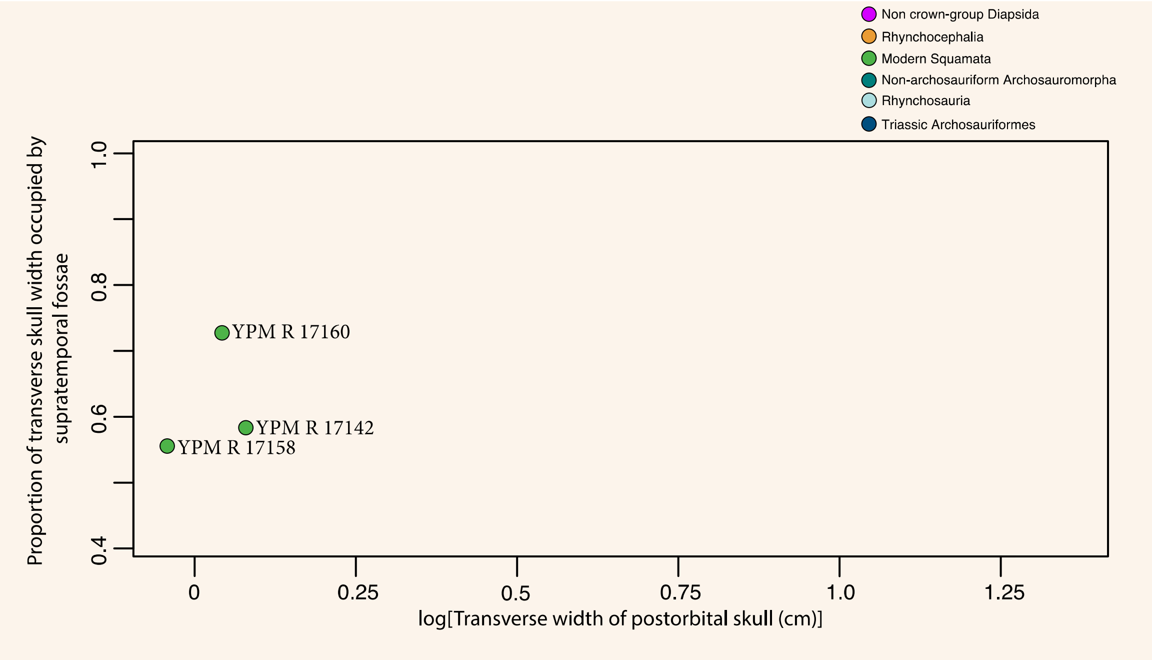

Supplementary Fig. 6. Bivariate plot of the transverse width of the postorbital portion of the skull (measured at the anteroposterior midpoints of the supratemporal fenestrae) against the proportional contribution of the supratemporal fossae to the transverse width of the postorbital portion of the skull. Plot illustrates *Sceloporus occidentalis* specimens sampled.

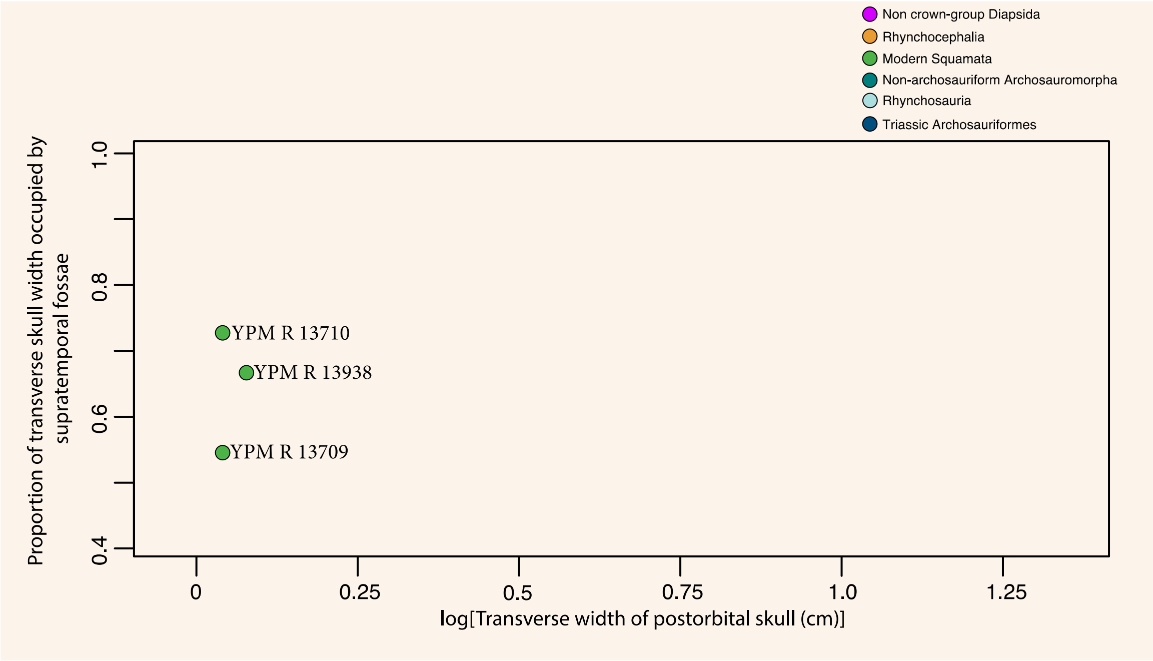

Supplementary Fig. 7. Bivariate plot of the transverse width of the postorbital portion of the skull (measured at the anteroposterior midpoints of the supratemporal fenestrae) against the proportional contribution of the supratemporal fossae to the transverse width of the postorbital portion of the skull. Plot illustrates *Teius teyou* specimens sampled.

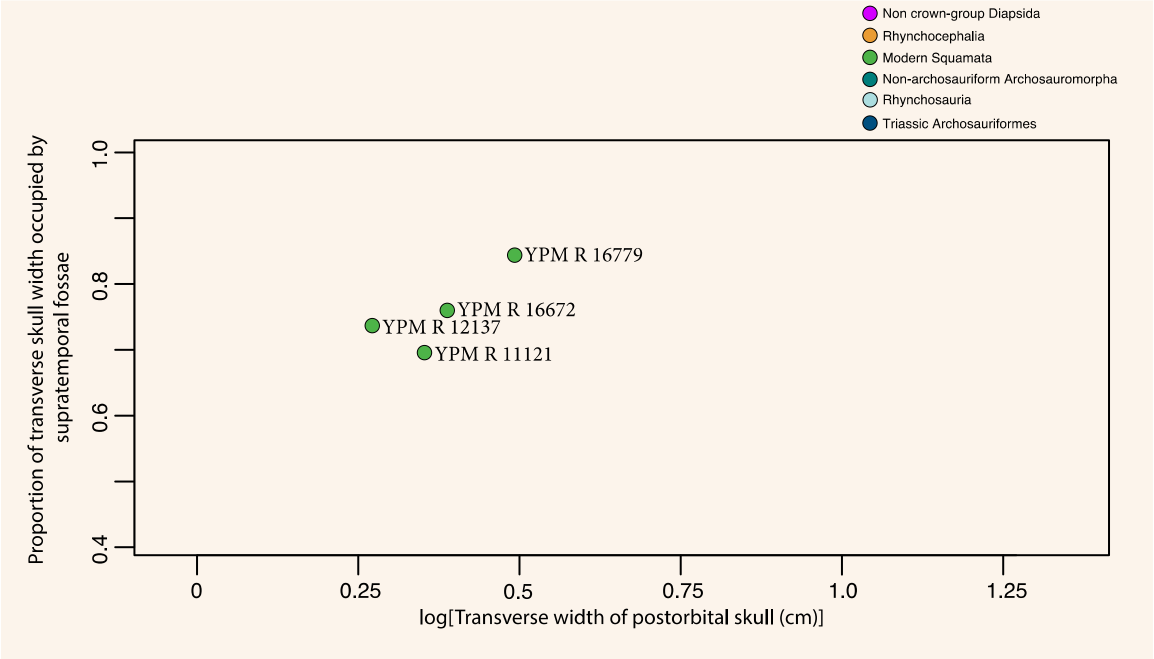

Supplementary Fig. 8. Bivariate plot of the transverse width of the postorbital portion of the skull (measured at the anteroposterior midpoints of the supratemporal fenestrae) against the proportional contribution of the supratemporal fossae to the transverse width of the postorbital portion of the skull. Plot illustrates *Physignathus lesueurii* specimens sampled.

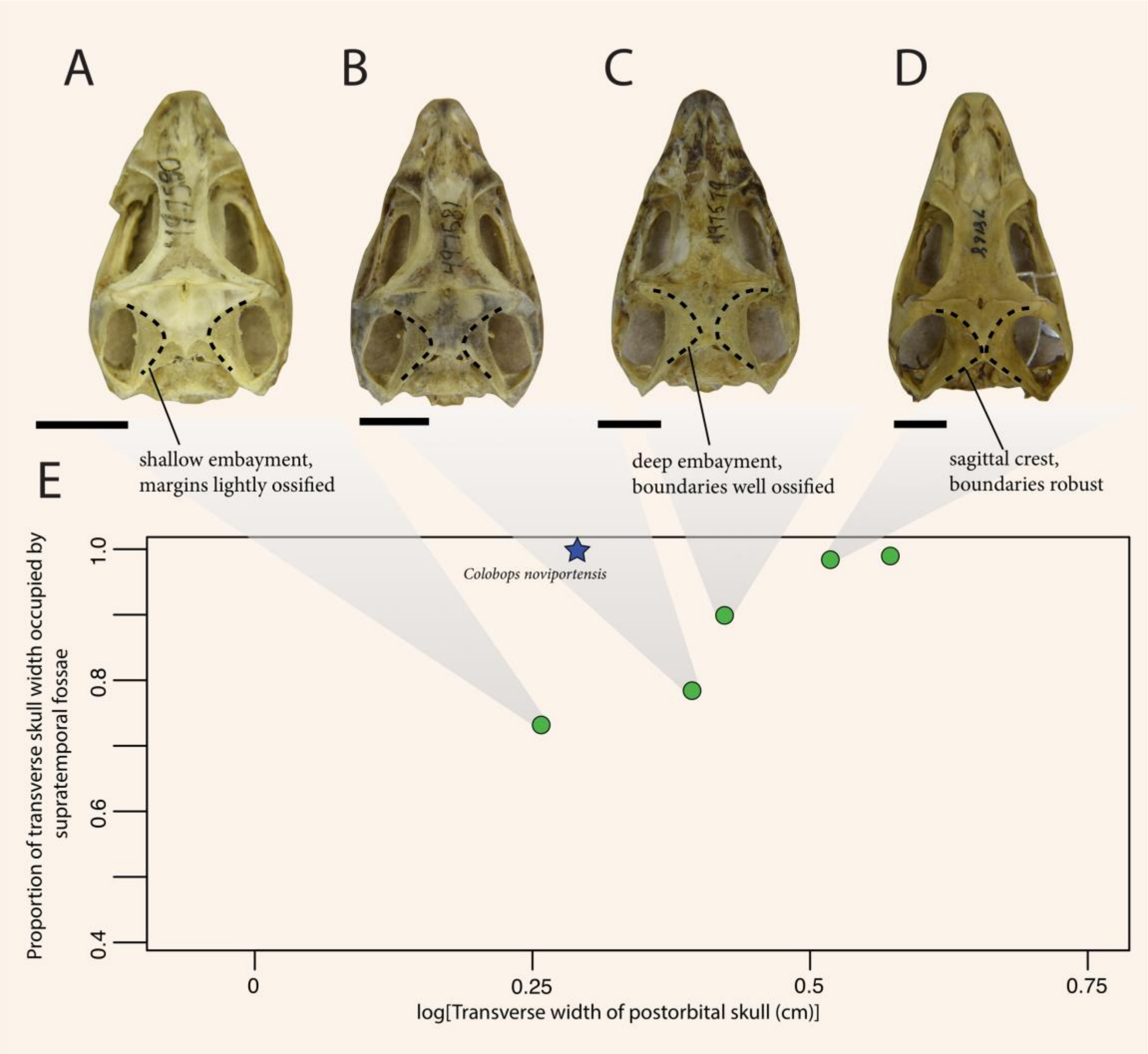

Supplementary Fig. 9. Skulls of *Hydrosaurus pustulatus* in dorsal view, illustrating the size-linked expansion of the supratemporal fossae: (A) USNM VZ 497580, (B) USNM VZ 498581, (C) USNM VZ 497579, (D) USNM VZ 78168 and (E) Bivariate plot of the transverse width of the postorbital portion of the skull (measured at the anteroposterior midpoints of the supratemporal fenestrae) against the proportional contribution of the supratemporal fossae to the transverse width of the postorbital portion of the skull in *Hydrosaurus pustulatus*. Shaded indicators indicate correspondence between data points and the photographs of the individual skulls. The star on the plot indicates the position of *Colobops noviportensis* (YPM VPPU 18835) in morphospace.

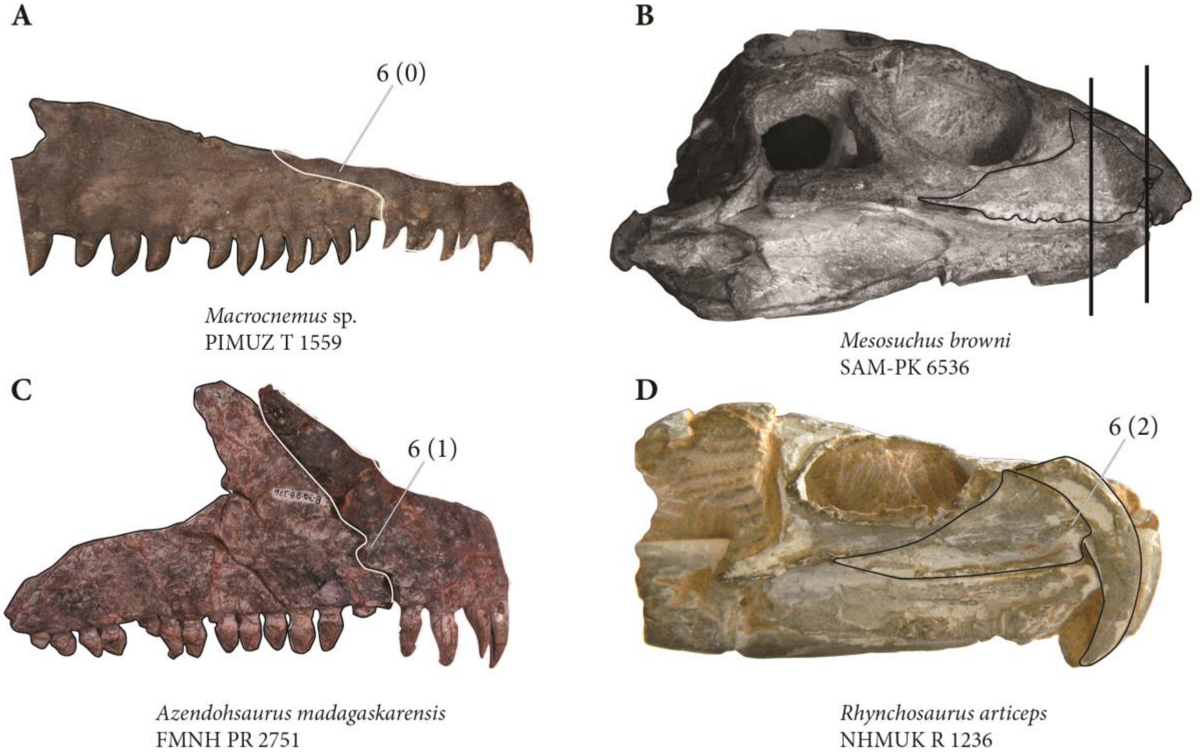

Supplementary Fig. 10. Illustration of the authors' interpretations of the states for character 6, describing the nature of the contact between premaxilla and maxilla in Archosauromorpha. Taxa illustrated include (A) *Macrocnemus* sp., (B) *Mesosuchus browni* (mirrored), (C) *Azendohsaurus madagaskarensis*, and (D) *Rhynchosaurus articeps* (mirrored). State 0 describes a straight suture, in which the premaxilla overlies the maxilla anterolaterally. State 1 describes an irregular suture, in which a small posterior "tab" on the premaxilla laps over the anterolateral surface of the maxilla. State 2 is newly introduced for this contribution, describing a condition in which the maxilla possesses an anterolaterally directed lamina that laps over the posterolateral surface of the premaxilla. This condition is not immediately apparent in (B), but it can be seen in CT scan data of the specimen. The vertical lines in (B) illustrate the position of CT slices illustrated in Supplementary Figure 11.

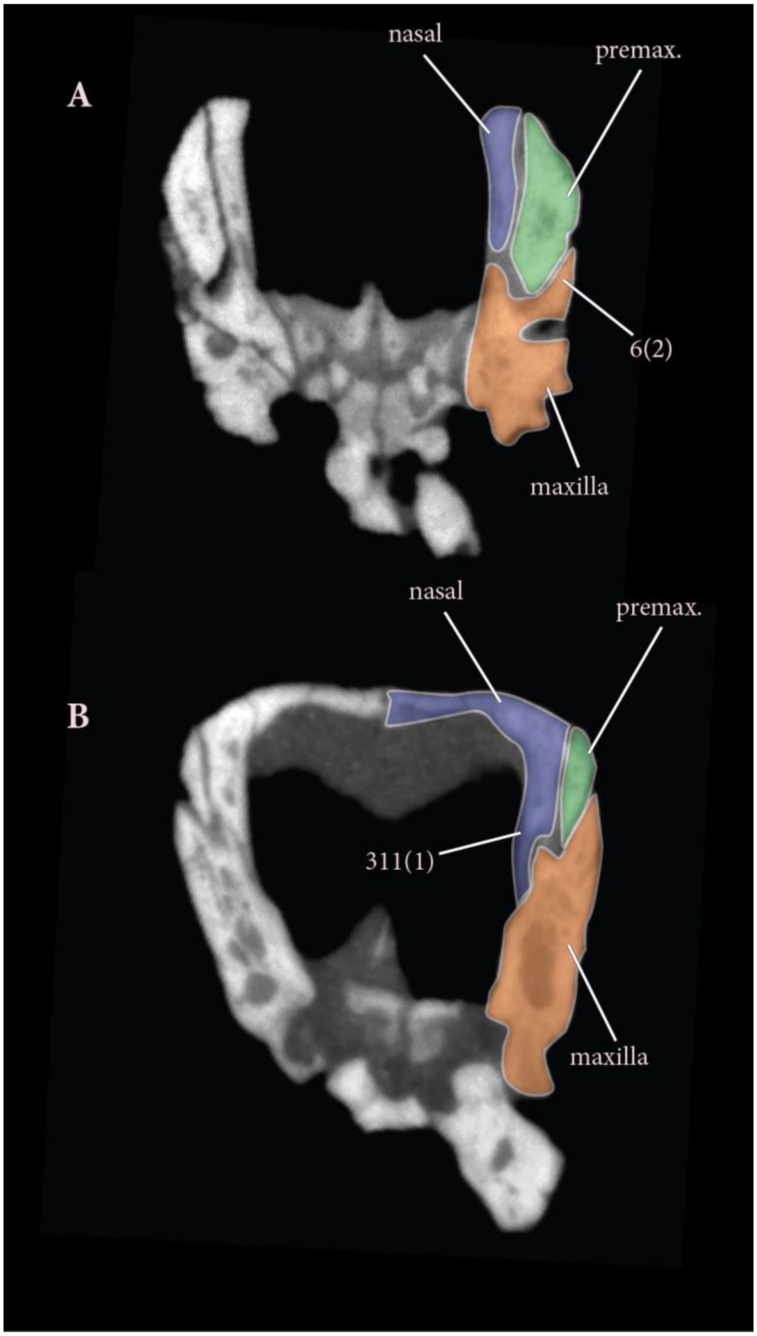

Supplementary Fig. 11. Coronal CT slices of the skull of *Mesosuchus browni* (SAM PK-6536), illustrating characters 6 and 311. (A) Slice 101/1122. (B) Slice 143/1122. Character 311 describes the prominence of the ventral lamina of the nasal bone, which laps ventrally across the dorsoventral extent of the dorsal process of the maxilla. Positions of slices on the specimen are illustrated in Supplemental Figure 10C.

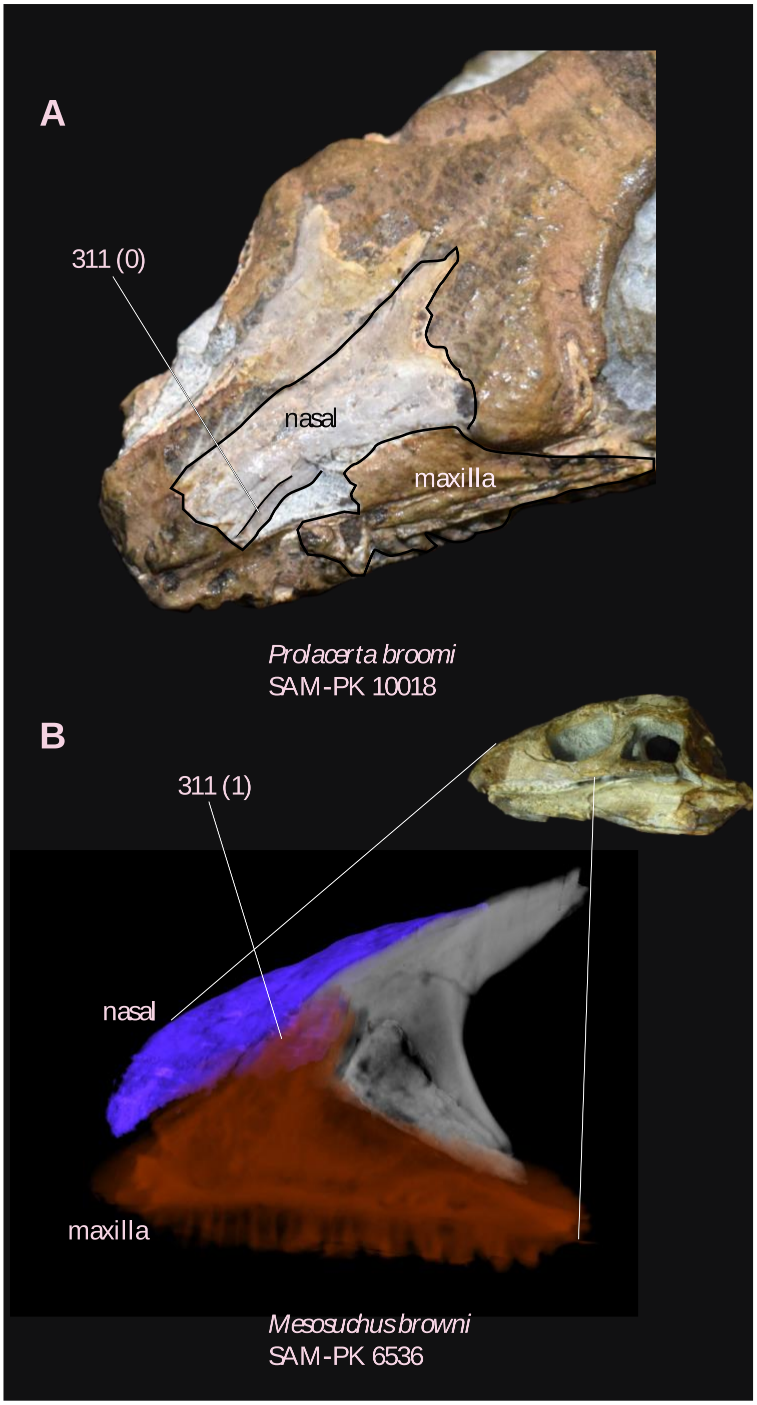

Supplementary Fig. 12. Illustration of the states for Character 311. (A) Illustration of state 0 with the rostrum of *Prolacerta broomi* in left anterolateral view. The edges of the left nasal and left maxilla have been outlined. (B) Illustration of a three-dimensional volume rendering of the nasal (purple), maxilla (orange), lacrimal (gray), and prefrontal (gray) based on µCT scan data of *Mesosuchus browni* in left lateral view, with a photograph of the skull indicating the position of these elements on the complete specimen. The maxilla has been rendered slightly transparent to show the depth of the ventral lamina of the nasal in lateral view.

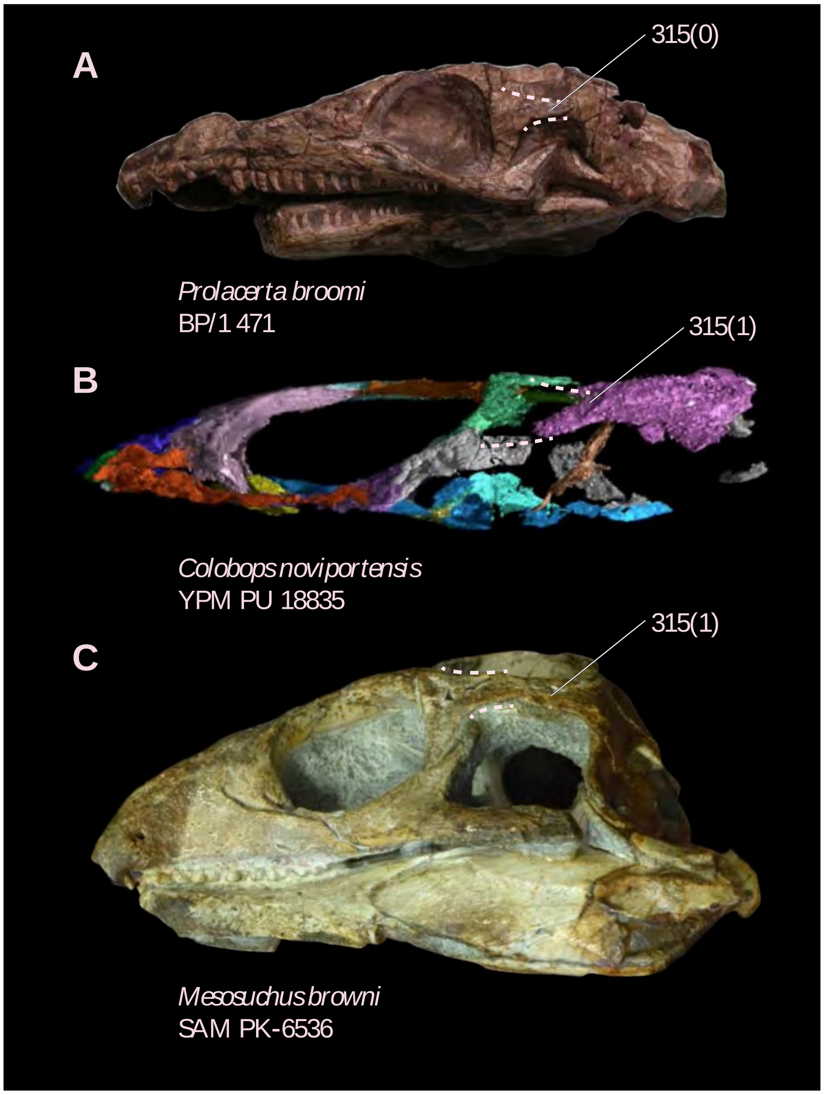

Supplementary Fig. 13. Illustration of states for Character 315, describing the position of the postorbital bar relative to the dorsal margin of the orbit. We consider the bar to have been positioned at or near the dorsal margin of the orbit due to (i) the height of the anterior process of the squamosal and its clear facet for the posterior process of the postorbital and (ii) the base of the posterior process of the postorbital (broken on both sides) being positioned near the dorsal margin of the orbit.

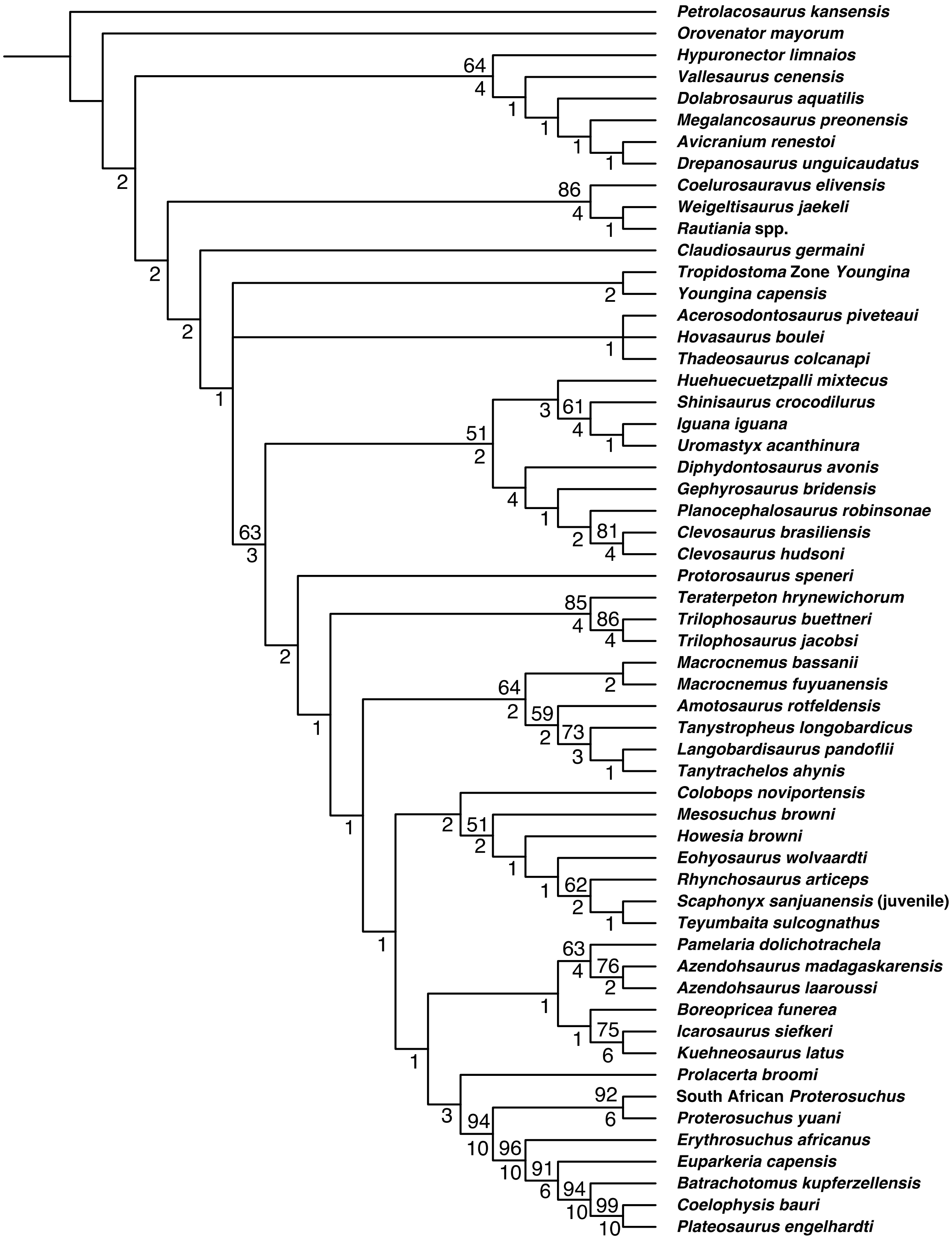

Supplementary Fig. 14. Strict consensus of most-parsimonious trees resulting from analysis in TNT v. 1.5. Numbers above nodes indicate frequency of nodes resulting from bootstrap resampling analysis. Numbers below nodes indicate Bremer support values.

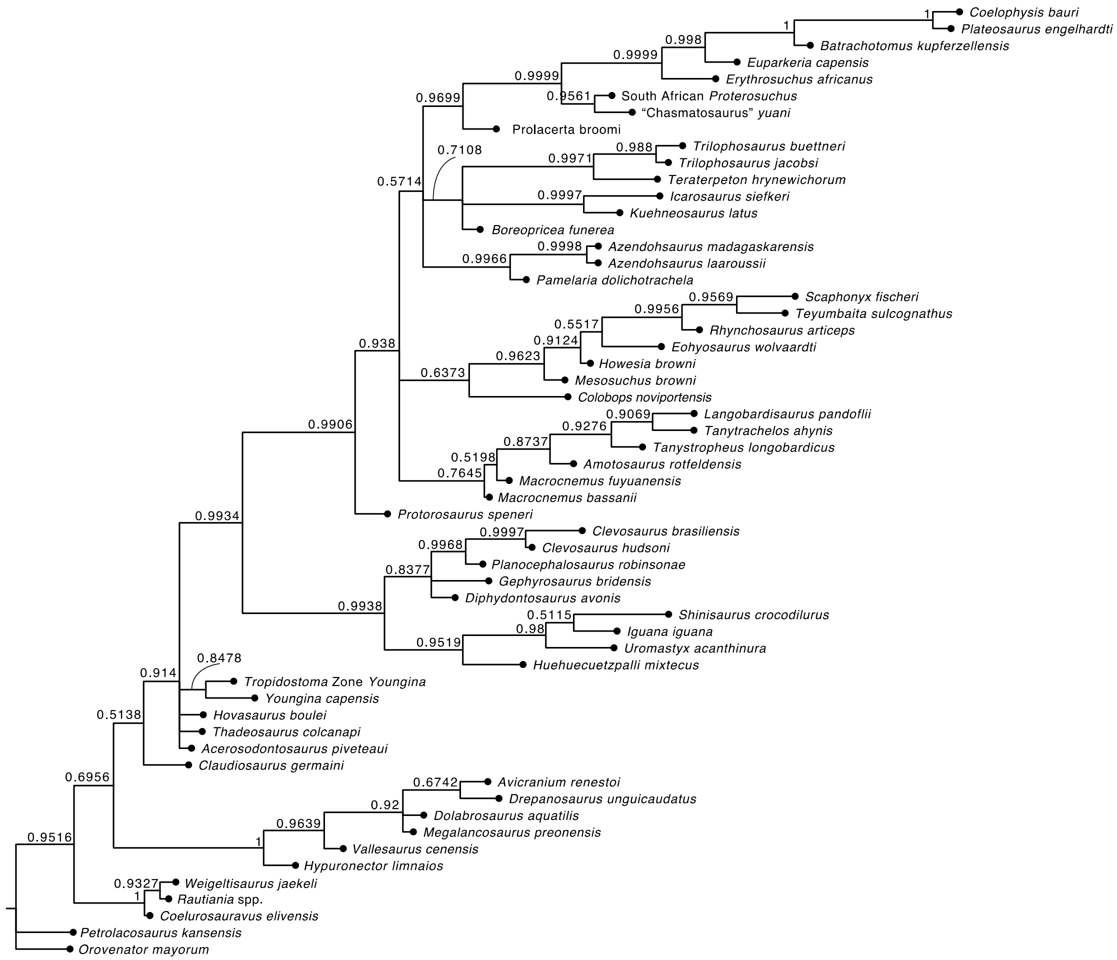

Supplementary Fig. 15. 50% majority rules topology resulting from Bayesian phylogenetic analysis performed in MrBayes 3.23. Numbers above branches indicate posterior probabilities of recovered clades.

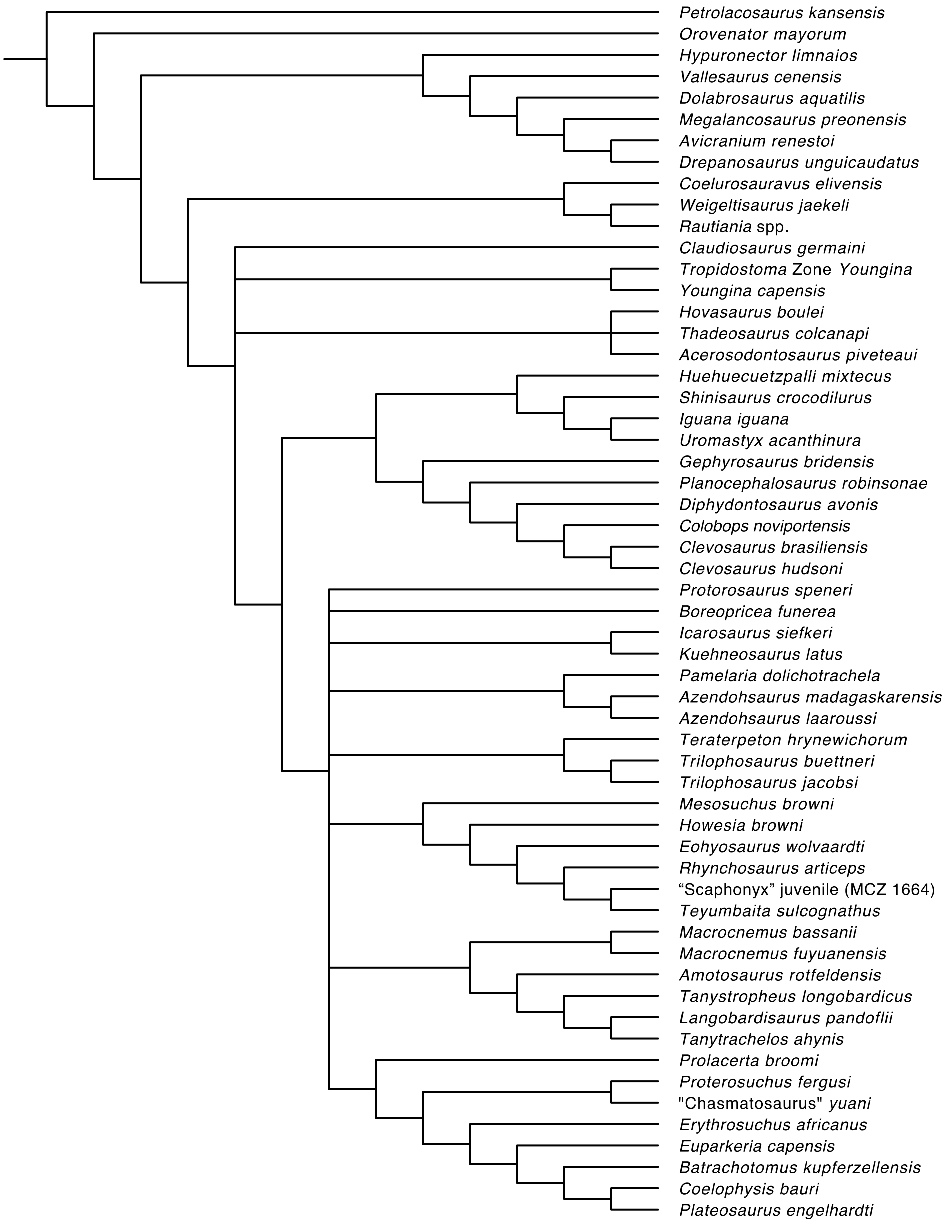

Supplementary Fig. 16. Strict consensus of most-parsimonious trees resulting from analysis in TNT v. 1.5. *Colobops noviportensis* was constrained to be in a clade with all Sphenodontia in this analysis. Parameters for this analysis can be found in Materials and Methods.

Supplemental Table – Measurements of skull dimensions

TAXON	SOURCE	STF Width at AP midpoint (cm)	STF Surface area (cm²)	Transverse width of postorbital skull (cm)	Antero-posterior length of skull (cm)	Triangle circumscribed by ½-width/length	Adductor Chamber / Total width
Azendohsaurus madagaskarensis	Flynn et al., 2010	3.053	-----	7.264	-----	-----	0.8405837
Bentonyx sidensis	Hone and Benton, 2010	2.909	12.046	6.046	12.851	19.4242865	0.962289117
Brasinorhynchus mariantensis	Schultz et al., 2016	9.87	72.726	19.96	32.053	159.94447	0.988977956
Clevosaurus brasiliensis	Hsiou et al., 2015	0.566	0.331	1.522	2.142	0.815031	0.743758213
Clevosaurus hudsoni	NHMUK PLR 605	0.303	0.122	0.948	2.024	0.479688	0.639240506
Ctenosaura similis	YPM R 10533	2.4	-----	5.3	-----	-----	0.905660377
Dracaena guiananensis	YPM R 12496	1.8	-----	4.3	-----	-----	0.837209302
Dracaena guianensis	YPM R 11075	0.6	-----	1.9	-----	-----	0.631578947
Dracaena guianensis	YPM R 11286	0.95	-----	2.6	-----	-----	0.730769231
Eohyosaurus woolvardti	Butler et al., 2015	1.594	-----	3.44	-----	-----	0.926744186
Euparkeria capensis	SAM-PK 5867 - CT scan data	1.094	1.074	2.912	8.63	6.28264	0.751373626
Gracilisuchus stipanicicorum	MCZ 4117 – CT scan data	1.538	1.353	4.146	7.987	8.2785255	0.741919923
Herrerasaurus ischigualastensis	PVSJ 407 - scan data	2.943	10.49	7.578	30	56.835	0.77672209
Hydrosaurus pustulatus	NMNH (herp.) 78168	1.59	1.667	3.24	5.116	4.14396	0.51054501
Hydrosaurus pustulatus	NMNH (herp.) 78170	1.91	2.88	3.88	6.714	6.51258	0.98453608
Hydrosaurus pustulatus	NMNH (herp.) 497579	1.15	0.882	2.58	4.024	2.59548	0.89147287
Hydrosaurus pustulatus	NMNH (herp.) 497580	0.66	0.435	1.78	2.916	1.29762	0.74157303
Hydrosaurus pustulatus	NMNH (herp.) 497581	0.913	0.697	2.34	3.721	2.176785	0.78034188
Iguana iguana	NMNH (herp.) 70470	2.55	4.685	5.12	8.789	11.24992	0.99609375
Iguana iguana	NMNH (herp.) 220231	1.54	1.712	3.36	5.69	4.7796	0.91666667
Iguana iguana	NMNH (herp.) 220232	0.40	0.25	1.54	2.203	0.848155	0.51948052

Iguana iguana	NMNH (herp.) 248781	0.73	0.553	2.00	2.974	1.487	0.73
Iguana iguana	NMNH (herp.) 220236	1.87	2.596	3.80	7.524	7.1478	0.98421053
Iguana iguana	YPM R 11188	0.45	-----	1.6	-----	-----	0.5625
Iguana iguana	YPM R 11622	1.65	-----	3.4	-----	-----	0.970588235
Iguana iguana	YPM R 18330	1.3	-----	2.8	-----	-----	0.928571429
Iguana iguana	YPM R 10869	2.75	-----	5.6	-----	-----	0.982142857
Melanorosaurus carinatus	Barrett and Yates, 2006	3.395	23.438	7.55	33.067	62.4139625	0.899337748
Mesosuchus browni	SAM-PK 6536	1.701	3.071	3.87	4.947	4.7862225	0.879069767
Petrolacosaurus kansensis	Reisz (1981) reconstruction	0.5	0.2	2.432	5.693	3.461344	0.411184211
Physignathus lesueurii	YPM R 11121	0.8	-----	2.3	-----	-----	0.695652174
Physignathus lesueurii	YPM R 12137	0.7	-----	1.9	-----	-----	0.736842105
Physignathus lesueurii	YPM R 16672	0.95	-----	2.5	-----	-----	0.76
Physignathus lesueurii	YPM R 16779	1.35	-----	3.2	-----	-----	0.84375
Pogona vitticeps	YPM R 16629	0.7	-----	2	-----	-----	0.7
Pogona vitticeps	YPM R 12139	1.2	-----	2.7	-----	-----	0.888888889
Prolacerta broomi	BP/1 471	0.891	0.818	2.674	6.073	4.0598005	0.666417352
Prolacerta broomi	NMQR 3763/1	1.161	-----	2.82	----	-----	0.823404255
Prolacerta broomi	NMQR 3763/2	1	0.967	2.364	4.837	2.858667	0.846023689
Prolacerta broomi	BP/1 5375	1.023	1.014	2.358	-----	-----	0.867684478
Proterosuchus alexanderi	NMQR 1484	2.198	6.932	5.996	20.85	31.25415	0.733155437
Proterosuchus fergusi	BP/1 4016	1.835	4.339	5.43	16.19	21.977925	0.67587477
Proterosuchus fergusi	BP/1 3393	4.298	12.263	10.39	20.85	69.9792475	0.827333975
Proterosuchus fergusi	RC 846 – CT scan data	3.016	9.794	8.826	-----	-----	0.683435305
Protorosaurus speneri	USNM cast of NMK	1.273	1.906	2.726	8.822	6.012193	0.933969186
Sauromalus varius	YPM R 19209	1.15	-----	3.5	-----	-----	0.657142857
Sauromalus varius	YPM R 19211	0.45	-----	1.8	-----	-----	0.5
Sceloporus occidentalis	YPM R 17158	0.25	-----	0.9	-----	-----	0.555555556
Sceloporus occidentalis	YPM R 17142	0.35	-----	1.2	-----	-----	0.583333333

Sceloporus occidentalis	YPM R 17160	0.4	-----	1.1	-----	-----	0.727272727
Shansisuchus shansisuchus	Fig. 2 of Young, 1964	5.826	-----	17.296	-----	-----	0.673681776
Sphenodon punctatum	Unnumbered specimen from California Academy of Sciences	1.573	-----	3.234	-----	-----	0.972789116
Sphenodon punctatum	YPM R 11431	1.15	-----	2.6	-----	-----	0.884615385
***Sphenodon punctatum* (hatchling)**	Digimorph images of CM 30660	0.2703	0.082	0.9812	1.294	0.3174182	0.550958011
***Sphenodon punctatum* (juvenile)**	Jones and Lappin (2009) illustration of NMNZRE 0156	0.7483	0.47	1.6784	2.593	1.0880228	0.891682555
Sphenodon puncatum	Digimorph images of YPM R 9194	1.708	2.88	3.622	5.519	4.9974545	0.943125345
Colobops noviportensis	YPM PU 18835	0.9191	0.661	1.8634	2.363	1.10080355	0.986476334
Tanystropheus longobardicus	PIMUZ/T 2819	2.602	4.986	5.396	16.98	22.90602	0.964418087
Teius teyou	YPM R 13709	0.3	-----	1.1	-----	-----	0.545454545
Teius teyou	YPM R 13938	0.4	-----	1.2	-----	-----	0.666666667
Teius teyou	YPM R 13710	0.4	-----	1.1	-----	-----	0.727272727
Teyujagua paradoxa	Pinheiro et al., 2016	1.854	3.384	4.636	9.271	10.745089	0.799827437
Teyumbaita sulcognathus	Montefeltro et al, 2010	9.18	55.471	18.45	19.951	92.0239875	0.995121951
Trilophosaurus buettneri	TMM 31025-140 (average of both sides because of preservational distortion)	2.35	11.495	4.82	-----	-----	0.975103734
Tropidostoma* Zone *Youngina	SAM-PK 6205	0.2625	0.078	1.05	2.000	0.525	0.5
Varanus exanthematicus	YPM R 11187	0.55	-----	2.1	-----	-----	0.523809524

Varanus exanthematicus	YPM R 19118	0.3	-----	1.2	-----	-----	0.5
Varanus exanthematicus	YPM R 13940	0.65	-----	2.3	-----	-----	0.565217391
Varanus komodoensis	YPM R 10881	2.8	-----	6.9	-----	-----	0.811594203
Varanus komodoensis	YPM R 16943	2.7	-----	6.9	-----	-----	0.782608696
Youngina capensis	SAM-PK 7578	0.416	0.271	1.424	4.083	1.453548	0.584269663
Youngina capensis	BP/1 70	0.489	0.364	1.63	-----	-----	0.6
Youngina capensis	BP/1 3859	0.548	0.296	1.808	-----	-----	0.60619469

Supplemental Table 1. Measurements of the transverse widths of the post-orbital portion of the skull at the anteroposterior midpoint of the supratemporal fenestra, the transverse width of the supratemporal fossae (here measured from the supratemporal fossae) at the same point, and the anteroposterior length of the skull from the anterior tip of the preserved rostrum to the posterior margin of the parietal. Measurements from fossil taxa were derived from the literature or from photographs by A. Pritchard. Measurement methods can be found in Appendix D.

Supplementary References

1. Case, G. R. in *A Pictorial Guide to Fossils* 514 (Van Nostrand Reinhold Company, 1982).
2. Sues, H.-D. & Baird, D. A skull of a sphenodontian lepidosaur from the New Haven Arkose (Upper Triassic: Norian) of Connecticut. *J. Vertebr. Paleontol.* **13,** 370–372 (1993).
3. Currie, P. J. A new younginid (Reptilia: Eosuchia) from the Upper Permian of Madagascar. *Can. J. Earth Sci.* **17,** 500–511 (1980).
4. Bickelmann, C., Müller, J. & Reisz, R. R. The enigmatic diapsid *Acerosodontosaurus piveteaui* (Reptilia: Neodiapsida) from the Upper Permian of Madagascar and the paraphyly of "younginiform" reptiles. *Can. J. Earth Sci.* **46,** 651–661 (2009).
5. Carroll, R. L. Plesiosaur ancestors from the Upper Permian of Madagascar. *Philos. Trans. R. Soc. Lond. B Biol. Sci.* **293,** 315–383 (1981).
6. Caldwell, M. W. Developmental constraints and limb evolution in Permian and extant lepidosauromorph diapsids. *J. Vertebr. Paleontol.* **14,** 459–471 (1994).
7. Evans, S. E. The gliding reptiles of the Upper Permian. *Zool. J. Linn. Soc.* **76,** 97–123 (1982).
8. Evans, S. E. & Haubold, H. A review of the Upper Permian genera *Coelurosauravus*, *Weigeltisaurus* and *Gracilisaurus* (Reptilia: Diapsida). *Zool. J. Linn. Soc.* **90,** 275–303 (1987).
9. Bulanov, V. V. & Sennikov, A. G. New data on the morphology of Permian gliding weigeltisaurid reptiles of Eastern Europe. *Paleontol. J.* **44,** 682–694 (2010).
10. Berman, D. S. & Reisz, R. R. *Dolabrosaurus aquatilis*, a small lepidosauromorph reptile from the Upper Triassic Chinle Formation of north-central New Mexico. *J. Paleontol.* 1001–1009 (1992).
11. Renesto, S., Spielmann, J. A., Lucas, S. G. & Spagnoli, G. T. The taxonomy and paleobiology of the Late Triassic (Carnian-Norian: Adamanian-Apachean) drepanosaurs (Diapsida: Archosauromorpha: Drepanosauromorpha): Bulletin 46. *Bull. N. M. Mus. Nat. Hist.* **46,** 1–81 (2010).
12. Pritchard, A. C., Turner, A. H., Irmis, R. B., Nesbitt, S. J. & Smith, N. D. Extreme Modification of the Tetrapod Forelimb in a Triassic Diapsid Reptile. *Curr. Biol.* **26,** 2779–2786 (2016).
13. Currie, P. J. *Hovasaurus boulei*, an aquatic eosuchian from the Upper Permian of Madagascar. *Palaeontol. Afr.* **24,** 99–168 (1981).
14. Colbert, E. H. & Olsen, P. E. A new and unusual aquatic reptile from the Lockatong Formation of New Jersey (Late Triassic, Newark Supergroup). *Am. Mus. Novit.* **3334,** 1–24 (2001).
15. Reisz, R. A diapsid reptile from the Pennsylvanian of Kansas. *Spec. Publ. Mus. Nat. Hist. Univ. Kans.* **7,** 1–74 (1981).
16. Bulanov, V. V. & Sennikov, A. G. The first gliding reptiles from the Upper Permian of Russia. *Paleontol. J.* **40,** S567–S570 (2006).
17. Currie, P. J. & Carroll, R. L. Ontogenetic changes in the eosuchian reptile *Thadeosaurus*. *J. Vertebr. Paleontol.* **4,** 68–84 (1984).
18. Smith, R. M. H. & Evans, S. E. An aggregation of juvenile *Youngina* from the Beaufort Group, Karoo Basin, South Africa. *Palaeontology* **39,** 289–303 (1996).
19. Piveteau, J. Paléontologie de Madasgascar XIII—amphibiens et reptiles Permiens. *Ann. Paléontol.* **15,** 55–178 (1926).

20. Bulanov, V. V. & Sennikov, A. G. Substantiation of validity of the Late Permian genus *Weigeltisaurus* Kuhn, 1939 (Reptilia, Weigeltisauridae). *Paleontol. J.* **49,** 1101–1111 (2015).
21. Goodrich, E. S. The hind foot of *Youngina* and fifth metatarsal in Reptilia. *J. Anat.* **76,** 308–312 (1942).
22. Gow, C. E. The morphology and relationships of *Youngina capensis* Broom and Prolacerta broomi Parrington. *Palaeontol. Afr.* **18,** 89–131 (1975).
23. Gardner, N. M., Holliday, C. M. & O'Keefe, F. R. The braincase of *Youngina capensis* (Reptilia, Diapsida): new insights from high-resolution CT scanning of the holotype. *Palaeontol. Electron.* **13,** 16 p. (2010).
24. Fraser, N. C. & Rieppel, O. A new protorosaur (Diapsida) from the Upper Buntsandstein of the Black Forest, Germany. *J. Vertebr. Paleontol.* **26,** 866–871 (2006).
25. Flynn, J. J., Nesbitt, S. J., Parrish, J. M., Ranivoharimanana, L. & Wyss, A. R. A new species of *Azendohsaurus* (Diapsida: Archosauromorpha) from the Triassic Isalo Group of southwestern Madagascar: cranium and mandible. *Palaeontology* **53,** 669–688 (2010).
26. Nesbitt, N., Sterling *et al.* Postcranial osteology of *Azendohsaurus madagaskarensis* (?Middle to Upper Triassic, Isalo Group of Madagascar) and its systematic position among stem archosaurs. *Bull. Am. Mus. Nat. Hist.* **398,** 1–126 (2015).
27. Hone, D. W. E. & Benton, M. J. A new genus of rhynchosaur from the Middle Triassic of south-west England. *Palaeontology* **51,** 95–115 (2008).
28. Langer, M. C., Montefeltro, F. C., Hone, D. E., Whatley, R. & Schultz, C. L. On Fodonyx spenceri and a new rhynchosaur from the Middle Triassic of Devon. *J. Vertebr. Paleontol.* **30,** 1884–1888 (2010).
29. Benton, M. J. & Allen, J. L. *Boreopricea* from the Lower Triassic of Russia, and the relationships of the prolacertiform reptiles. *Palaeontology* **40,** 931–954 (1997).
30. Schultz, C. L., Langer, M. C. & Montefeltro, F. C. A new rhynchosaur from south Brazil (Santa Maria Formation) and rhynchosaur diversity patterns across the Middle-Late Triassic boundary. *PalZ* **90,** 593–609 (2016).
31. Dilkes, D. W. The rhynchosaur *Howesia browni* from the Lower Triassic of South Africa. *Palaeontology* **38,** 665–685 (1995).
32. Benton, M. J. & Kirkpatrick, R. Heterochrony in a fossil reptile: juveniles of the rhynchosaur *Scaphonyx fischeri* from the Late Triassic of Brazil. *Palaeontology* **32,** 335–353 (1989).
33. Colbert, E. H. The Triassic gliding reptile *Icarosaurus*. *Bull. Am. Mus. Nat. Hist.* **143,** 85–142 (1970).
34. Robinson, P. L. Gliding lizards from the Upper Keuper of Great Britain. *Proc. Geol. Soc. Ldon* **1601,** 137–146 (1962).
35. Robinson, P. L. Triassic vertebrates from lowland and upland. *Sci. Cult.* **33,** 169–173 (1967).
36. Saller, F., Renesto, S. & Dalla Vecchia, F. M. First record of *Langobardisaurus* (Diapsida, Protorosauria) from the Norian (Late Triassic) of Austria, and a revision of the genus. *Neues Jahrb. Für Geol. Paläontol.-Abh.* **268,** 83–95 (2013).
37. Peyer, B. Die Triasfauna der Tessiner Kalkalpen XII. *Macrocnemus bassanii* Nopcsa. *Abh. Schweiz. Palaeontol. Ges.* **59,** 1–140 (1937).
38. Rieppel, O. The hind limb of *Macrocnemus bassanii* (Nopcsa)(Reptilia, Diapsida): development and functional anatomy. *J. Vertebr. Paleontol.* **9,** 373–387 (1989).

39. Li, C., Zhao, L. & Wang, L. A new species of *Macrocnemus* (Reptilia: Protorosauria) from the Middle Triassic of southwestern China and its palaeogeographical implication. *Sci. China Ser. Earth Sci.* **50,** 1601–1605 (2007).
40. Dilkes, D. W. The Early Triassic rhynchosaur *Mesosuchus browni* and the interrelationships of basal archosauromorph reptiles. *Philos. Trans. R. Soc. B Biol. Sci.* **353,** 501–541 (1998).
41. Sen, K. *Pamelaria dolichotrachela*, a new prolacertid reptile from the Middle Triassic of India. *J. Asian Earth Sci.* **21,** 663–681 (2003).
42. Ezcurra, M. D. The phylogenetic relationships of basal archosauromorphs, with an emphasis on the systematics of proterosuchian archosauriforms. *PeerJ* **4,** e1778 (2016).
43. Gottmann-Quesada, A. & Sander, P. M. A redescription of the early archosauromorph *Protorosaurus speneri* MEYER, 1832, and its phylogenetic relationships. *Palaeontogr. Abt. A* 123–220 (2009).
44. Benton, M. J. The species of *Rhynchosaurus*, a rhynchosaur (Reptilia, Diapsida) from the Middle Triassic of England. *Philos. Trans. R. Soc. Lond. B Biol. Sci.* **328,** 213–306 (1990).
45. Ezcurra, M. D., Montefeltro, F. & Butler, R. J. The early evolution of rhynchosaurs. *Front. Ecol. Evol.* **3,** 142 (2016).
46. Wild, R. Die Triasfauna der Tessiner XXIII: *Tanystropheus longobardicus* (Bassani). *Schweiz. Paläontol. Abh.* **95,** 1–162 (1973).
47. Wild, R. Die Triasfauna der Tessiner Kalkalpen XXIV. Neue Funde von *Tanystropheus* (Reptilia, Squamata). *Schweiz. Paläontol. Abh.* **102,** 1–43 (1980).
48. Olsen, P. E. A new aquatic eosuchian from the Newark Supergroup (Late Triassic–Early Jurassic) of North Carolina and Virginia. *Postilla* **176,** 1–14 (1979).
49. Pritchard, A. C., Turner, A. H., Nesbitt, S. J., Irmis, R. B. & Smith, N. D. Late Triassic tanystropheids (Reptilia, Archosauromorpha) from northern New Mexico (Petrified Forest Member, Chinle Formation) and the biogeography, functional morphology, and evolution of Tanystropheidae. *J. Vertebr. Paleontol.* **35,** e911186 (2015).
50. Sues, H.-D. An unusual new archosauromorph reptile from the Upper Triassic Wolfville Formation of Nova Scotia. *Can. J. Earth Sci.* **40,** 635–649 (2003).
51. Pinheiro, F. L., França, M. A., Lacerda, M. B., Butler, R. J. & Schultz, C. L. An exceptional fossil skull from South America and the origins of the archosauriform radiation. *Sci. Rep.* **6,** (2016).
52. Montefeltro, F. C., Langer, M. C. & Schultz, C. L. Cranial anatomy of a new genus of hyperodapedontine rhynchosaur (Diapsida, Archosauromorpha) from the Upper Triassic of Southern Brazil. *Earth Environ. Sci. Trans. R. Soc. Edinb.* **101,** 27–52 (2010).
53. Montefeltro, F. C., Bittencourt, J. S., Langer, M. C. & Schultz, C. L. Postcranial anatomy of the hyperodapedontine rhynchosaur *Teyumbaita sulcognathus* (Azevedo and Schultz, 1987) from the Late Triassic of southern Brazil. *J. Vertebr. Paleontol.* **33,** 67–84 (2013).
54. Gregory, J. T. Osteology and relationships of *Trilophosaurus*. *Univ. Tex. Publ.* **4401,** 273–359 (1945).
55. Parks, P. Cranial anatomy and mastication of the Triassic reptile *Trilophosaurus*. (University of Texas, Austin, 1969).
56. Spielmann, J. A., Lucas, S. G., Rinehart, L. F. & Heckert, A. B. The Late Triassic archosauromorph *Trilophosaurus*. *Bull. N. M. Mus. Nat. Hist.* **43,** 1–177 (2008).

57. Gower, D. J. The cranial and mandibular osteology of a new rauisuchian archosaur from the Middle Triassic of southern Germany. *Stuttg. Beitr. Zur Naturkunde Ser. B* **280,** 1–49 (1999).
58. Gower, D. J. Braincase evolution in suchian archosaurs (Reptilia: Diapsida): evidence from the rauisuchian *Batrachotomus kupferzellensis*. *Zool. J. Linn. Soc.* **136,** 49–76 (2002).
59. Gower, D. J. & Schoch, R. R. Postcranial anatomy of the rauisuchian archosaur *Batrachotomus kupferzellensis*. *J. Vertebr. Paleontol.* **29,** 103–122 (2009).
60. Romer, A. S. The Chanares (Argentina) Triassic reptile fauna XI. Two new long-snouted thecodonts, *Chanaresuchus* and *Gualosuchus*. *Breviora* **379,** 1–22 (1971).
61. Romer, A. S. The Chanares (Argentina) Triassic reptile fauna. XIII. An early ornithosuchid pseudosuchian, *Gracilisuchus stipanicicorum*, gen. et sp. nov. *Breviora* **389,** 1–24 (1972).
62. Young, C. C. On a new *Chasmatosaurus* from Sinkiang. *Bull. Geol. Soc. China* **15,** 291–311 (1936).
63. Young, C. C. Additional remains of *Chasmatosaurus yuani* Young from Sinkiang, China. *Vertebr. Palasiat.* **7,** 215–222 (1963).
64. Colbert, E. H. The Triassic dinosaur *Coelophysis*. *Bull. Mus. North. Ariz.* **57,** 1–160 (1989).
65. Nesbitt, S. J. The early evolution of archosaurs: relationships and the origin of major clades. *Bull. Am. Mus. Nat. Hist.* **352,** 1–292 (2011).
66. Gower, D. J. The braincase of the early archosaurian reptile *Erythrosuchus africanus*. *J. Zool.* **242,** 557–576 (1997).
67. Gower, D. J. The tarsus of erythrosuchid archosaurs, and implications for early diapsid phylogeny. *Zool. J. Linn. Soc.* **116,** 347–375 (1996).
68. Gower, D. J. Osteology of the early archosaurian reptile *Erythrosuchus africanus* Broom. *Ann. South Afr. Mus.* **110,** 1–88 (2003).
69. Ewer, R. F. The Anatomy of the Thecodont Reptile *Euparkeria capensis* Broom. *Philos. Trans. R. Soc. Lond. B Biol. Sci.* **248,** 379–435 (1965).
70. Senter, P. New information on cranial and dental features of the Triassic archosauriform reptile *Euparkeria capensis*. *Palaeontology* **46,** 613–621 (2003).
71. Yates, A. M. The first complete skull of the Triassic dinosaur *Melanorosaurus* Haughton (Sauropodomorpha: Anchisauria). *Spec. Pap. Palaeontol.* 9–55 (2007).
72. Galton, P. & Upchurch, P. Prosauropoda. in *The Dinosauria, 2nd edition* (eds. Weishampel, D. B., Dodson, P. & Osmolska, H.) 232–258 (University of California Press, 2004).
73. Prieto-Márquez, A. & Norell, M. A. Redescription of a nearly complete skull of *Plateosaurus* (Dinosauria: Sauropodomorpha) from the Late Triassic of Trossingen (Germany). *Am. Mus. Novit.* **3727,** 1–58 (2011).
74. Fabbri, M. *et al.* The skull roof tracks the brain during the evolution and development of reptiles including birds. *Nat. Ecol. Evol.* **1,** 1543 (2017).
75. Cruickshank, A. R. I. The proterosuchian thecodonts. in *Studies in vertebrate evolution* (eds. Joysey, K. A. & Kemp, T. S.) 89–119 (Oliver & Boyd, 1972).
76. Ezcurra, M. D. & Butler, R. J. Taxonomy of the proterosuchid archosauriforms (Diapsida: Archosauromorpha) from the earliest Triassic of South Africa, and implications for the early archosauriform radiation. *Palaeontology* **58,** 141–170 (2015).
77. Young, C.-C. The pseudosuchians in China. *Palaeontol. Sin.* **151,** 1–205 (1964).
78. Arantes, B. de A., Soares, M. B. & Schultz, C. L. *Clevosaurus brasiliensis* (Lepidosauria, Sphenodontia) do Triássico Superior do Rio Grande do Sul: anatomia pós-craniana e relaçoes filogenéticas. *Rev. Bras. Paleontol.* **12,** 43–54 (2009).

79. Hsiou, A. S., De França, M. A. G. & Ferigolo, J. New Data on the *Clevosaurus* (Sphenodontia: Clevosauridae) from the Upper Triassic of Southern Brazil. *PloS One* **10,** e0137523 (2015).
80. Fraser, N. C. The osteology and relationships of *Clevosaurus* (Reptilia: Sphenodontida). *Philos. Trans. R. Soc. Lond. B Biol. Sci.* **321,** 125–178 (1988).
81. Fraser, N. C. & Shelton, C. G. Studies of tooth implantation in fossil tetrapods using high-resolution X-radiography. *Geol. Mag.* **125,** 117–122 (1988).
82. Whiteside, D. I. The head skeleton of the Rhaetian sphenodontid *Diphydontosaurus avonis* gen. et sp. nov. and the modernizing of a living fossil. *Philos. Trans. R. Soc. Lond. B Biol. Sci.* **312,** 379–430 (1986).
83. Evans, S. E. The skull of a new eosuchian reptile from the Lower Jurassic of South Wales. *Zool. J. Linn. Soc.* **70,** 203–264 (1980).
84. Evans, S. E. Tooth replacement in the Lower Jurassic lepidosaur *Gephyrosaurus bridensis*. *Neues Jahrb. Für Geol. Paläontol. Monatshefte* **7,** 411–420 (1985).
85. Evans, S. E. The postcranial skeleton of the Lower Jurassic eosuchian *Gephyrosaurus bridensis*. *Zool. J. Linn. Soc.* **73,** 81–116 (1981).
86. Fraser, N. C. A new rhynchocephalian from the British Upper Trias. *Palaeontology* **25,** 709–725 (1982).
87. Fraser, N. C. & Walkden, G. M. The postcranial skeleton of the Upper Triassic sphenodontid *Planocephalosaurus robinsonae*. *Palaeontology* **27,** 575–595 (1984).
88. Conrad, J. L. Skull, mandible, and hyoid of *Shinisaurus crocodilurus* Ahl (Squamata, Anguimorpha). *Zool. J. Linn. Soc.* **141,** 399–434 (2004).
89. Conrad, J. L. Postcranial skeleton of *Shinisaurus crocodilurus* (Squamata: Anguimorpha). *J. Morphol.* **267,** 759–775 (2006).
90. Carroll, R. L. A pleurosaur from the Lower Jurassic and the taxonomic position of the Sphenodontida. *Palaeontogr. Abt. A* **189,** 1–28 (1985).
91. Evans, S. E. The skull of lizards and tuatara. in *Biology of the Reptilia. Morphology H. The Skull of Lepidosauria* (ed. Gans, C.) **20,** 1–347 (2008).
92. Günther, A. Contribution to the anatomy of *Hatteria* (Rhynchocephalus, Owen). *Philos. Trans. R. Soc. Lond.* **157,** 595–629 (1867).
93. Hoffstetter, R. & Gasc, J.-P. Vertebrae and ribs of modern reptiles. in *Biology of the Reptilia. Morphology A.* (ed. Gans, C.) **1,** 201–310 (Academic Press, 1969).
94. Howes, G. B. & Swinnerton, H. H. On the development of the skeleton of the Tuatara. *Trans. Zool. Soc. Lond.* **16,** 1–85 (1901).
95. Miner, R. W. The pectoral limb of *Eryops* and other primitive tetrapods. *Bull. Am. Mus. Nat. Hist.* **51,** 145–308 (1925).
96. Rénous-Lécuru, Sabine. Morophologie comparée du carpe chez des Lepidosauriens actuels (Rhynchocéphales, Lacertiliens, Amphisbéniens). *Gegenbaurs Morphol. Jahrb.* **119,** 727–766 (1973).
97. Jones, M. E. H., Curtis, N., Fagan, M. J., O'Higgins, P. & Evans, S. E. Hard tissue anatomy of the cranial joints in *Sphenodon* (Rhynchocephalia): sutures, kinesis, and skull mechanics. *Palaeontol. Electron.* **14,** 1–92 (2011).
98. Jones, M. E. & Lappin, A. K. Bite-force performance of the last rhynchocephalian (Lepidosauria: Sphenodon). *J. R. Soc. N. Z.* **39,** 71–83 (2009).

99. Ketcham, R. A. *Sphenodon punctatus* (tuatara) - juvenile. (1999). Available at: http://digimorph.org/specimens/Sphenodon_punctatus/juvenile/. (Accessed: 1st November 2017)
100. Pritchard, A. C. & Nesbitt, S. J. A bird-like skull in a Triassic diapsid reptile increases heterogeneity of the morphological and phylogenetic radiation of Diapsida. *R. Soc. Open Sci.* **4,** 170499 (2017).
101. Vaughn, P. P. The Permian reptile *Araeoscelis* restudied. *Bull. Mus. Comp. Zool.* **113,** 305–467 (1955).
102. El-Toubi, M. R. The post-cranial osteology of the lizard, *Uromastyx aegyptia* (Forska al). *J. Morphol.* **84,** 281–292 (1949).
103. Ezcurra, M. D., Scheyer, T. M. & Butler, R. J. The origin and early evolution of Sauria: reassessing the Permian saurian fossil record and the timing of the crocodile-lizard divergence. *PloS One* **9,** e89165 (2014).
104. Evans, S. E., Prasad, G. V. R. & Manhas, B. K. Rhynchocephalians (Diapsida: Lepidosauria) from the Jurassic Kota Formation of India. *Zool. J. Linn. Soc.* **133,** 309–334 (2001).
105. Butler, R. J., Ezcurra, M. D., Montefeltro, F. C., Samathi, A. & Sobral, G. A new species of basal rhynchosaur (Diapsida: Archosauromorpha) from the early Middle Triassic of South Africa, and the early evolution of Rhynchosauria. *Zool. J. Linn. Soc.* **174,** 571–588 (2015).
106. Jones, M. E. H. *et al.* Integration of molecules and new fossils supports a Triassic origin for Lepidosauria (lizards, snakes, and tuatara). *BMC Evol. Biol.* **13,** (2013).
107. Oelrich, T. M. The anatomy of the head of *Ctenosaura pectinata* (Iguanidae). *Misc. Publ. Univ. Mich. Mus. Zool.* **94,** 1–122 (1956).
108. Evans, S. E. A new lizard-like reptile (Diapsida: Lepidosauromorpha) from the Middle Jurassic of England. *Zool. J. Linn. Soc.* **103,** 391–412 (1991).
109. Rieppel, O. & Gronowski, R. W. The loss of the lower temporal arcade in diapsid reptiles. *Zool. J. Linn. Soc.* **72,** 203–217 (1981).
110. Gauthier, J. A., Estes, R. & de Queiroz, K. A phylogenetic analysis of Lepidosauromorpha. in *Phylogenetic Relationships of the Lizard Families* 15–98 (Stanford University Press, 1988).
