# Supplementary material for: A tiny Triassic saurian from Connecticut and the early evolution of the diapsid feeding apparatus
Source: Nat Commun. 2018 Mar 23;9:1213. doi: 10.1038/s41467-018-03508-1 (PMC5865133; doi:10.1038/s41467-018-03508-1)
Supplement: Supplementary file 3 — Description of Additional Supplementary Files(PDF 52 kb) [file 41467_2018_3508_MOESM3_ESM.pdf]

Description of Additional Supplementary Files

File Name: Supplementary Movie 1
Description: Three-dimensional volume rendering of the holotype of *Colobops noviportensis* (YPM VPPU 18835). Skull bones are individually segmented and volume rendered in VG Studio Max 3.0.

File Name: Supplementary Movie 2
Description: Reconstructed CT slices from scan of YPM VPPU 18835 in an anteroposterior axis.

File Name: Supplementary Movie 3
Description: Reconstructed CT slices scan of YPM VPPU 18835 in a dorsoventral axis.

File Name: Supplementary Movie 4
Description: Reconstructed CT slices scan of YPM VPPU 18835 in a transverse axis.

File Name: Supplementary Movie 5
Description: Dynamic cutaway of three-dimensional volume rendering of scan of YPM VPPU 18835 in an anteroposterior axis.

File Name: Supplementary Movie 6
Description: Dynamic cutaway of three-dimensional volume rendering of scan of YPM VPPU 18835 in a dorsoventral axis.

File Name: Supplementary Movie 7
Description: Dynamic cutaway of three-dimensional volume rendering of scan of YPM VPPU 18835 in a transverse axis.
